# Nox4 reprograms cardiac substrate metabolism via protein O-GlcNAcylation to enhance stress adaptation

**DOI:** 10.1172/jci.insight.96184

**Published:** 2017-12-21

**Authors:** Adam A. Nabeebaccus, Anna Zoccarato, Anne D. Hafstad, Celio X.C. Santos, Ellen Aasum, Alison C. Brewer, Min Zhang, Matteo Beretta, Xiaoke Yin, James A. West, Katrin Schröder, Julian L. Griffin, Thomas R. Eykyn, E. Dale Abel, Manuel Mayr, Ajay M. Shah

**Affiliations:** 1 Cardiovascular Division, King’s College London British Heart Foundation Centre of Excellence, London, United Kingdom.; 2 Cardiovascular Research Group, Department of Medical Biology, UiT The Arctic University of Norway, Tromsø, Norway.; 3 Department of Biochemistry and Cambridge Systems Biology Centre, University of Cambridge, Cambridge, United Kingdom.; 4 Institut für Kardiovaskuläre Physiologie, Goethe-Universität, Frankfurt am Main, Germany.; 5 Division of Imaging Sciences & Biomedical Engineering, King’s College London British Heart Foundation Centre of Excellence, London, United Kingdom.; 6 Department of Medicine and Fraternal Order of Eagles Diabetes Research Center, Carver College of Medicine, University of Iowa, Iowa City, Iowa, USA.

**Keywords:** Cardiology, Metabolism, Cardiovascular disease, Intermediary metabolism, Signal transduction

## Abstract

Cardiac hypertrophic remodeling during chronic hemodynamic stress is associated with a switch in preferred energy substrate from fatty acids to glucose, usually considered to be energetically favorable. The mechanistic interrelationship between altered energy metabolism, remodeling, and function remains unclear. The ROS-generating NADPH oxidase-4 (Nox4) is upregulated in the overloaded heart, where it ameliorates adverse remodeling. Here, we show that Nox4 redirects glucose metabolism away from oxidation but increases fatty acid oxidation, thereby maintaining cardiac energetics during acute or chronic stresses. The changes in glucose and fatty acid metabolism are interlinked via a Nox4-ATF4–dependent increase in the hexosamine biosynthetic pathway, which mediates the attachment of O-linked N-acetylglucosamine (O-GlcNAcylation) to the fatty acid transporter CD36 and enhances fatty acid utilization. These data uncover a potentially novel redox pathway that regulates protein O-GlcNAcylation and reprograms cardiac substrate metabolism to favorably modify adaptation to chronic stress. Our results also suggest that increased fatty acid oxidation in the chronically stressed heart may be beneficial.

## Abstract 2

Nox4 reprograms intermediary metabolism in the heart through an ATF4-mediated enhancement of protein O-GlcNAcylation, and the resulting switch to increased fatty acid oxidation protects the overloaded heart.

## Introduction

The heart responds to chronic stresses such as hemodynamic overload through hypertrophic remodeling (i.e., ventricular hypertrophy), which initially maintains normal contractile function but, when protracted, progresses to heart failure (1). Pathological left ventricular hypertrophy (LVH) is characterized by a broad range of structural and functional perturbations including cardiomyocyte hypertrophy, abnormal excitation-contraction coupling, contractile dysfunction, altered myocyte viability, interstitial fibrosis, and arrhythmia. A prominent feature of LVH is an alteration in cardiac energy substrate utilization. Whereas the healthy adult heart relies predominantly on fatty acid oxidation for energy generation, there is a switch in substrate preference from fatty acids to carbohydrates in diverse animal models of LVH and heart failure (2–4). It is suggested that this switch away from fatty acid oxidation may be adaptive by increasing cardiac efficiency in terms of oxygen consumption, since glucose oxidation has a lower oxygen requirement than fatty acid oxidation. In line with this idea, therapeutic approaches that inhibit fatty acid oxidation and/or promote an increase in glucose utilization are currently being assessed in patients with chronic heart failure (4, 5). However, the exact relationship between changes in energy substrate utilization and the functional state of the hypertrophied heart or its propensity to develop heart failure remains unclear. Moreover, the mechanisms underlying these changes in energy metabolism are incompletely understood.

The complex changes in gene and protein expression and function that drive hypertrophic cardiac remodeling are directed by intracellular signaling pathways, which are activated in response to hemodynamic and other stresses (6, 7). A well-established paradigm in the field is that adaptive remodeling may be driven by molecular signaling pathways that are distinct from those that drive maladaptive remodeling. In this regard, redox-regulated signaling mediated by NADPH oxidase (Nox) family proteins is of particular interest (8). These membrane-associated enzymes generate ROS by catalyzing electron transfer from NADPH to molecular oxygen and are involved in localized redox signaling during processes such as cell proliferation, migration, differentiation, and tissue repair (9, 10). Of the 7 mammalian Nox isoforms (Nox1–5 and Duox1–2), Nox2 and Nox4 are expressed in the heart, and previous work from our laboratory and others identified distinct roles for these isoforms in the cardiac adaptation to stress conditions (9, 11). Nox2-mediated signaling promotes several detrimental processes in the pathologically remodeling heart, including cardiomyocyte hypertrophy, contractile dysfunction, arrhythmia, interstitial fibrosis, cell death, and cardiac rupture after myocardial infarction (11, 12). In contrast, gain- and loss-of-function approaches in mouse models showed that the upregulation of Nox4 during chronic hemodynamic or ischemic stress can mediate protective effects (13, 14). In the setting of chronic hemodynamic overload, Nox4 promoted adaptive cardiac remodeling, with less LVH and better preserved function (13). While a number of signaling pathways modulated by Nox4 that may protect the heart have been identified (13, 15, 16), the precise changes induced in the remodeling heart to promote compensated cardiac function remain incompletely understood.

To elucidate processes that mediate Nox4-dependent protection against pressure overload–induced adverse cardiac remodeling, we initially pursued a proteomics approach in which normal mouse hearts were compared with hearts from mice with cardiomyocyte-targeted Nox4 overexpression or cardiomyocyte-targeted Nox2 overexpression, with and without chronic hemodynamic stress. This revealed significant perturbations in metabolic pathways in the Nox4-modulated cardiac proteome as compared with the other groups. Subsequent detailed analyses revealed that Nox4 induces a substantial reprogramming of glucose and fatty acid metabolism. We find that Nox4 induces a marked increase in activity of the hexosamine biosynthetic pathway (HBP), a branch pathway of glycolysis that drives enhanced protein O-linked glycosylation with N-acetylglucosamine (O-GlcNAcylation), whereas there is a reduction in glucose oxidation through the TCA cycle. In parallel, fatty acid oxidation is significantly increased, such that the hearts maintain a well-compensated energetic status in response to chronic hemodynamic or acute neurohumoral stresses. Mechanistically, the Nox4-induced augmentation of HBP activity is mediated by the transcription factor ATF4, with the increase in fatty acid oxidation related at least in part to an increased O-GlcNAcylation of the fatty acid transporter CD36. Collectively, these findings uncover a Nox4-regulated pathway that drives interlinked and complementary changes in cardiac glucose and fatty acid utilization. This reprogramming of cardiac metabolism, which fully maintains energetic status, may be fundamental to the protective effects of Nox4 during chronic hemodynamic stress.

## Results

### Nox4 modulates the metabolic proteome of the mouse heart.

To obtain an overview of potential adaptive pathways triggered by Nox4, we first undertook a proteomic analysis by difference in-gel electrophoresis (DIGE) of hearts isolated from Nox4 TG mice and their WT littermates, both under unstressed conditions and in the early stages after induction of pressure overload. Nox4 TG mice were reported previously to have a modest increase in Nox4 levels and oxidase activity, comparable with the increase in Nox4 observed after chronic pressure overload in WT mice (13) (Supplemental Figure 1A; supplemental material available online with this article; https://doi.org/10.1172/jci.insight.96184DS1). They exhibited significantly less cardiac hypertrophy and better contractile function than WT littermates at 3 weeks after abdominal aortic banding (Supplemental Figure 1A). To control for potential nonspecific effects related to ROS production, we also studied the proteome profile of Nox2-overexpressing hearts (17) under similar conditions, therefore enabling isoform-specific pathways to be assessed.

**Figure 1:**
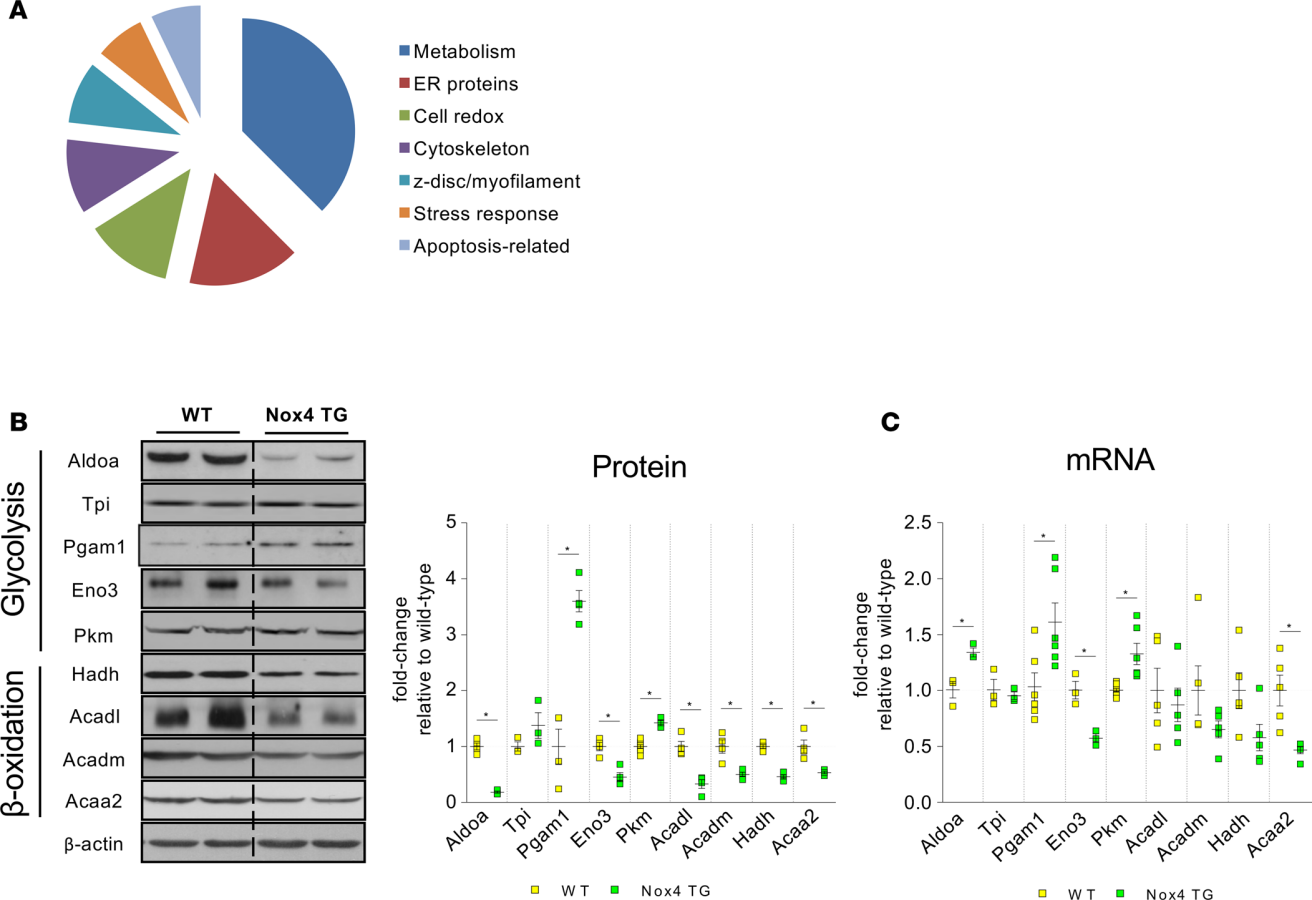
Nox4 alters the levels of cardiac proteins involved in glycolysis and fatty acid oxidation. (**A**) Categorization of differentially expressed proteins in Nox4 TG hearts by biological function, according to gene ontology. Difference in-gel electrophoresis (DIGE) was used to determine differentially expressed protein spots from WT and Nox4 TG hearts, followed by mass spectrometry to identify the proteins. (**B**) Immunoblotting for proteins involved in glycolysis and fatty acid β-oxidation. Representative immunoblots are shown to the left. Relative change in levels compared with WT hearts are shown to the right. *P < 0.05, 2-tailed *t* test (*n* = 3–4). See Supplemental Figure 1C for full names of the proteins. (**C**) mRNA expression levels of the proteins shown in **C**, expressed as fold-change compared with WT. *P < 0.05, 2-tailed *t* test (*n* = 3–6 per group).

A principal components analysis revealed that Nox4 TG hearts had a distinct proteome profile as compared with either WT or Nox2-overexpressing hearts, both under unstressed and stressed conditions (Supplemental Figure 1B). Unstressed Nox4 TG hearts had 97 significantly upregulated and 64 significantly downregulated protein spots as compared with WT littermates (Supplemental Figure 1C). Following protein identification by mass spectrometry (MS) and categorization according to gene ontology (www.geneontology.org), it was evident that Nox4 altered the expression level of many protein classes pertinent to heart failure pathobiology (Figure 1A). Of note, the category with the highest number of differentially expressed proteins was “metabolism.” Using Reactome analysis, among the most overrepresented pathways in Nox4 TG hearts were those involved in glucose and fatty acid metabolism (Table 1), with both up- and downregulation of different glycolytic proteins and a general downregulation of proteins involved in β-oxidation (Supplemental Figure 1D). Similar changes were also observed in the comparison of pressure-overloaded Nox4 TG versus WT hearts (Supplemental Figure 1D). We confirmed the pattern of changes in protein abundance in Nox4 TG hearts by IB (Figure 1B). The quantification of mRNA expression levels of these proteins revealed a similar pattern of change for most but not all transcripts (Figure 1C). Notably, most of the proteins involved in β-oxidation were not significantly altered at transcript level, apart from *Acaa2*. These findings suggest that glucose and fatty acid metabolism may be significantly perturbed by an increase in cardiomyocyte Nox4 levels.

### Nox4-overexpressing hearts exhibit altered substrate utilization.

**Figure 2:**
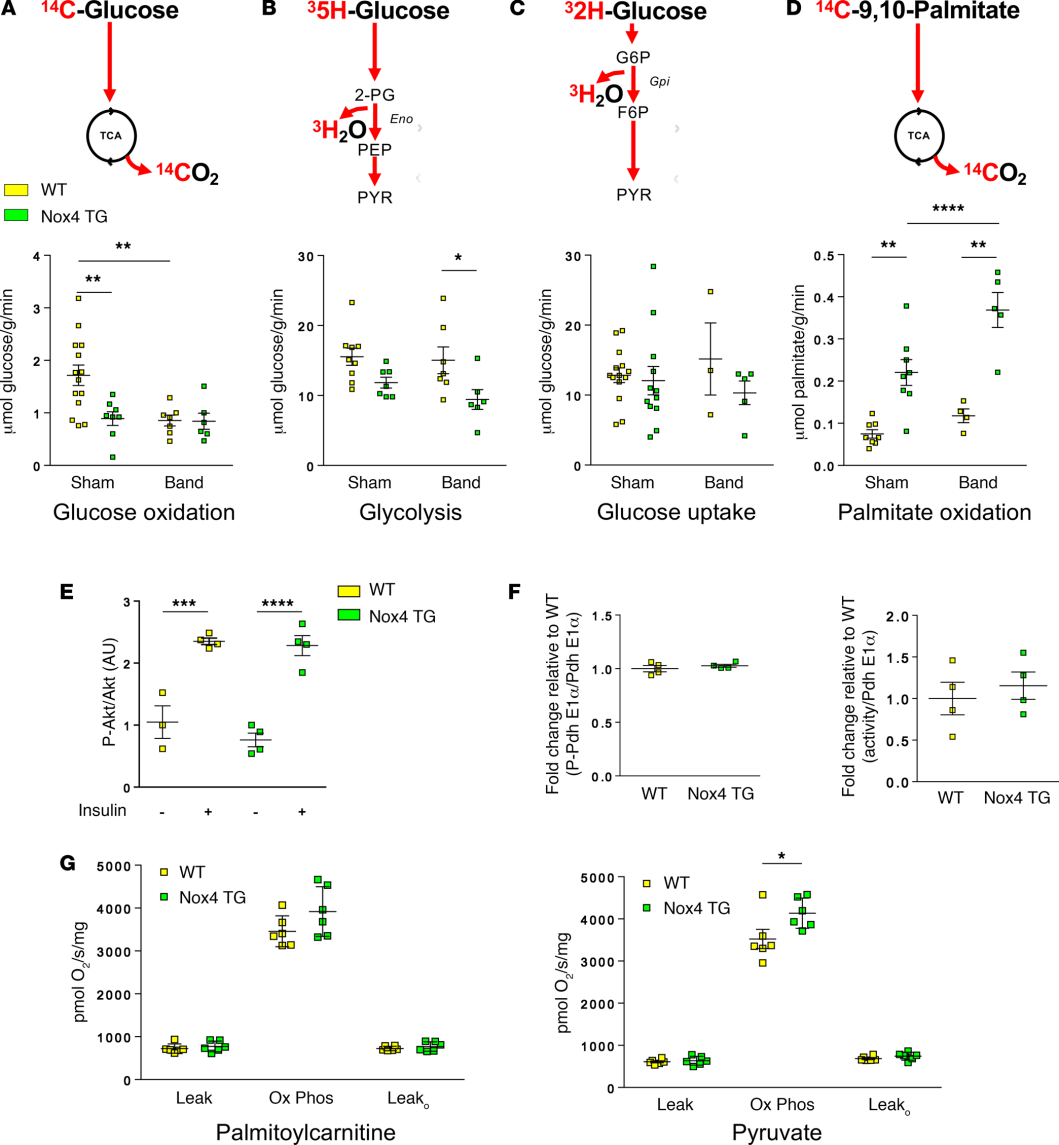
Myocardial substrate utilization in isolated Nox4 TG and WT hearts. (**A–D**) The schematics at the top show the tracers and readouts that were used. (**A**) Glucose oxidation rate (*n* = 6–14 per group); (**B**) glycolytic rate (*n* = 6–9 per group); (**C**) glucose uptake rate (*n* = 3–14 per group); and (**D**) palmitate oxidation rate (*n* = 4–8 per group). *P < 0.05, **P < 0.01, ****P < 0.0001, 2-way ANOVA with Tukey’s post hoc correction. (**E**) Myocardial insulin sensitivity assessed as the increase in Akt phosphorylation in response to i.p. insulin (0.75 IU/kg). The level of phosphorylated Akt is normalized by total Akt levels.***P < 0.001, ****P < 0.0001, 2-way ANOVA with Tukey’s post hoc correction (*n* = 3–4 per group). (**F**) Levels of phosphorylated pyruvate dehydrogenase (P-Pdha E1α) (left) and PDH activity (right) in mitochondrial fractions from WT and Nox4 TG hearts; 2-tailed *t* test, *n* = 4 per group. (**G**) Respiration with palmitoylcarnitine or pyruvate as substrates in mitochondria isolated from Nox4 TG and WT hearts. Leak denotes respiration measured in the presence of substrates and without ADP. Ox Phos denotes oxidative phosphorylation following the addition of ADP. Leak_o_ denotes respiration rates after addition of oligomycin. *P < 0.05, 2-tailed *t* test, *n* = 6 per group.

To ascertain the overall impact of the above changes on glucose and/or fatty acid utilization, we next performed ex vivo working heart perfusion studies with radiolabeled substrates to estimate glucose uptake, glycolysis, glucose oxidation, and fatty acid oxidation rates (18). Unstressed Nox4 TG hearts showed a significant decrease in glucose oxidation rate as compared with WT hearts. Glycolysis, as assessed by the production of ^3^H_2_O predominantly at the step catalyzed by enolase, also tended to be lower in Nox4 TG hearts (Figure 2, A and B), while the glucose uptake rate was unaltered (Figure 2C). In parallel, there was a profound increase in the fatty acid oxidation rate in Nox4 TG hearts (Figure 2D). Similar findings were observed in hypertrophied Nox4 TG hearts compared with hypertrophied WT controls (Figure 2, A–D).

There was no difference in insulin sensitivity between Nox4 TG and WT hearts, as assessed by an insulin-induced increase in myocardial Akt phosphorylation at Ser^473^ (Figure 2E and Supplemental Figure 2A). The increase in fatty acid oxidation in Nox4 TG hearts could, in principle, lead to an increased phosphorylation and inhibition of pyruvate dehydrogenase (PDH), thereby limiting the entry of glucose-derived pyruvate into the TCA cycle and reducing the glucose oxidation rate. However, we found no differences in PDH phosphorylation or activity between Nox4 TG and WT hearts (Figure 2F and Supplemental Figure 2B). We also assessed whether the changes in substrate utilization might be driven by alterations at the mitochondrial level. Mitochondria were isolated from Nox4 TG and WT hearts, and the respiration rates for different substrates were compared (Figure 2G). There was no difference in O_2_ consumption rate between genotypes with palmitoylcarnitine as a substrate, while the rate was slightly higher in the Nox4 TG group in the presence of pyruvate. These results indicate that the mitochondrial capacity for respiration is unaltered in Nox4 TG hearts and that the reduced rate of glucose oxidation and increased rate of fatty acid oxidation most likely occur independently of changes in mitochondrial metabolic capacity.

### Nox4 promotes an increase in proximal glycolytic metabolites and diversion into the HBP.

**Figure 3:**
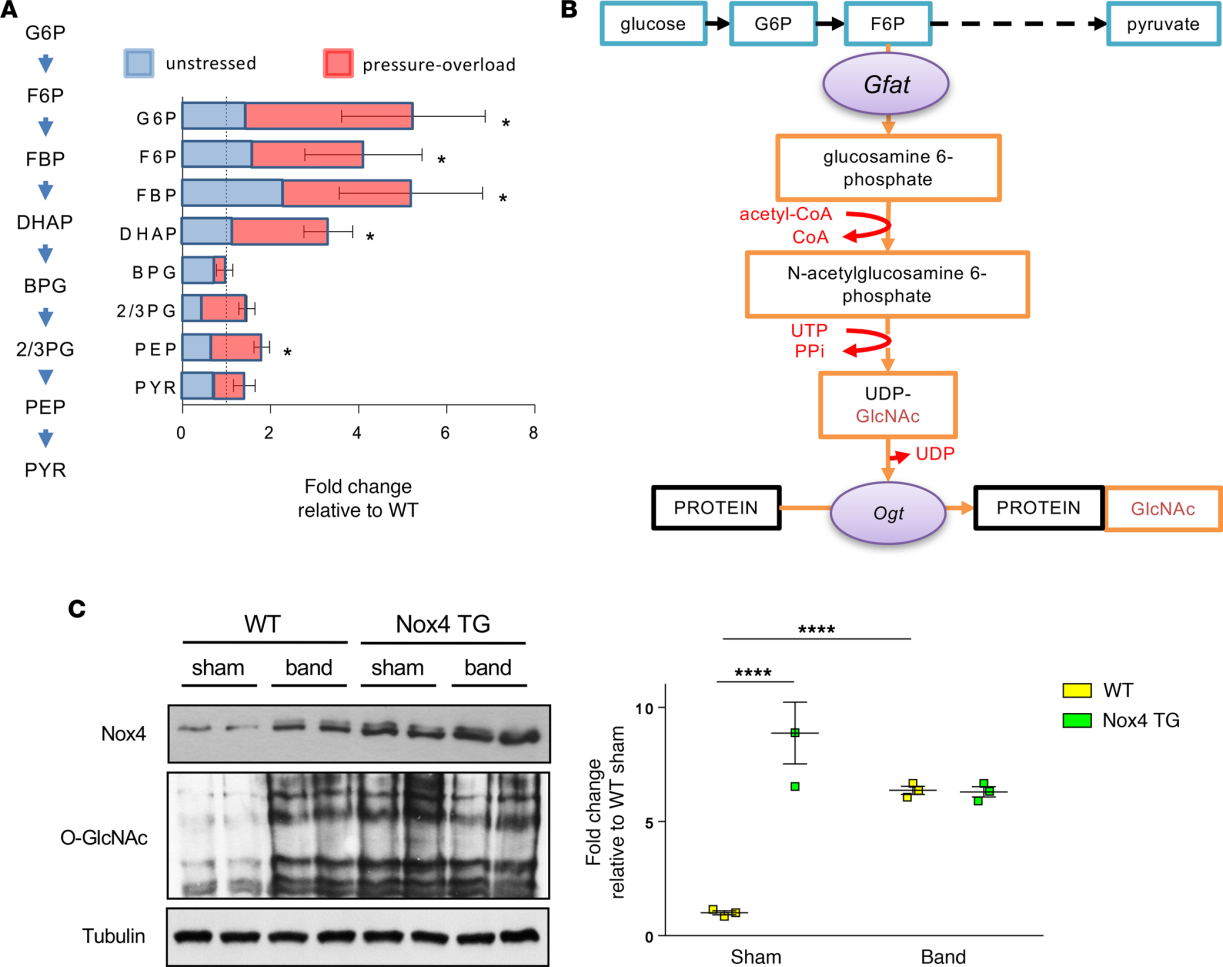
Nox4 enhances protein O-GlcNAcylation via the hexosamine biosynthetic pathway. (**A**) Glycolytic intermediates in Nox4 TG and WT hearts, quantified by LC-MS. Data are expressed as fold-change relative to WT. *P < 0.05, 2-tailed *t* test, *n* = 6. Schematic to the left shows the pathway for conversion of glucose 6-phosphate (G6P) to pyruvate (PYR). F6P, fructose 6-phosphate; FBP, fructose 1.6-bisphosphate; DHAP, dihydroxyacetone phosphate; BPG, 1,3-bisphosphoglycerate; 2,3PG, 3-phosphoglycerate and 2-phosphoglycerate; PEP, phosphoenolpyruvate. (**B**) Schematic of the hexosamine biosynthesis pathway (orange arrows), which branches off at F6P. Gfat, glutamine fructose-6-phosphate aminotransferase; Ogt, O-GlcNAc transferase. (**C**) Immunoblotting for O-GlcNAc–modified proteins in hearts of WT and Nox4 TG mice. Representative blots are shown to the left, and mean data are shown to the right. Coomassie staining of gels was used to confirm equal protein loading. ****P < 0.0001, 2-way ANOVA with Tukey’s post hoc correction; *n* = 3 per group.

The decrease in glycolysis and glucose oxidation in the absence of reduced glucose uptake in Nox4 TG hearts raised the possibility that the normal metabolism of glucose via a series of glycolytic reactions to pyruvate is perturbed. We therefore undertook targeted metabolomics by MS to quantify the levels of glycolytic intermediates in Nox4 TG and WT hearts. This analysis revealed that there was a relative increase in the steady-state pool of proximal glycolytic metabolites in unstressed Nox4 TG compared with WT hearts, and it was even more marked in hearts from animals that had undergone aortic banding (Figure 3A).

We surmised that the altered balance between proximal and distal glycolytic metabolites in Nox4 TG hearts might reflect increased diversion into branch pathways. One such pathway is the HBP, which metabolises fructose 6-phosphate to uridine diphosphate–N-acetylglucosamine (UDP-GlcNAc) (Figure 3B) and is known to be activated during the cardiac stress response to pressure overload (19). UDP-GlcNAc is utilized as the substrate for the posttranslational O-linked glycosylation (O-GlcNAcylation) of a wide variety of proteins whereby a single N-acetylglucosamine (O-GlcNAc) moiety is attached to serine and threonine residues by the enzyme O-GlcNAc transferase (Ogt) (20). Using an antibody that detects O-GlcNAc modifications, we found that overall protein O-GlcNAcylation was significantly increased in Nox4 TG hearts compared with WT, both under unstressed conditions and after chronic pressure overload (Figure 3C). These results indicate that Nox4 promotes a relative increase in glucose diversion into the HBP rather than the main pathway of glycolysis to pyruvate.

### Nox4 modulates fatty acid oxidation via the HBP.

**Figure 4:**
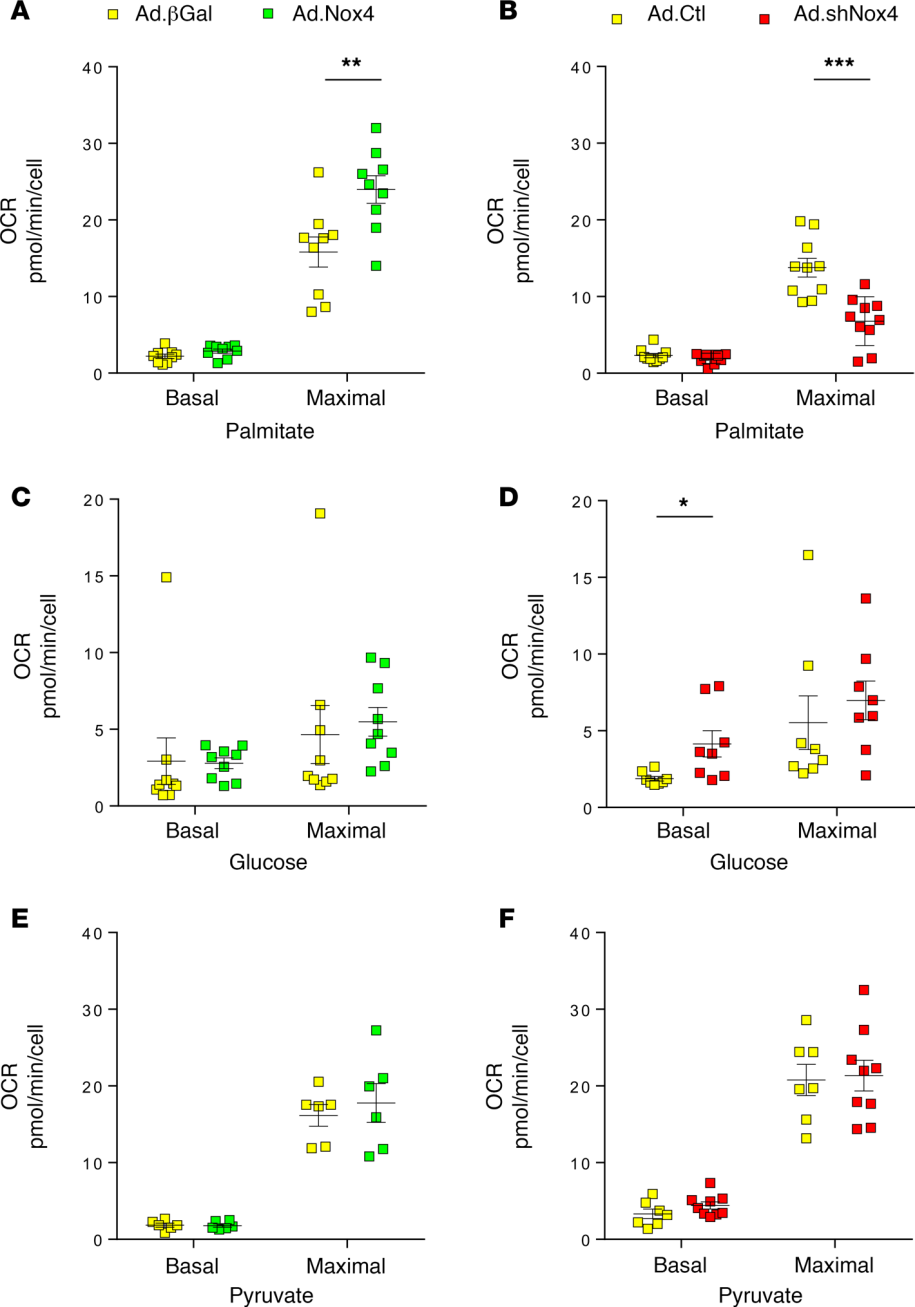
Nox4 increases fatty acid oxidation in cultured cardiomyocytes. (**A**, **C**, and **E**) Effect of adenoviral-mediated overexpression of Nox4 (Ad.Nox4) or a control β-galactosidase protein (Ad.β-Gal) on the oxygen consumption rate (OCR) in the presence of (**A**) palmitate, (**C**) glucose, or (**E**) pyruvate as substrate. (**B**, **D**, and **F**) Effect of shRNA-mediated knockdown of Nox4 (Ad.shNox4) or control (Ad.Ctl) on the OCR in the presence of (**B**) palmitate, (**D**) glucose, or (**F**) pyruvate as substrate. OCR was measured in an extracellular flux analyzer under basal conditions (Basal) and after the addition of FCCP (p-trifluoromethoxy carbonyl cyanide phenyl hydrazine) to induce maximal respiration (Maximal). *P < 0.05, **P < 0.01, ***P < 0.001, 2-way ANOVA with Sidak’s post hoc correction; *n* = 9 per group. See also Supplemental Figure 3.

To gain further insight into the relationship between Nox4 and altered substrate utilization, we turned to a cultured cardiomyocyte system in which either endogenous Nox4 was knocked down by infecting cells with an adenoviral vector expressing a specific shRNA or Nox4 levels were increased by infection with a Nox4 adenovirus (Supplemental Figure 3A). We studied the oxidation of different substrates with the use of an extracellular flux analyzer. The overexpression of Nox4 significantly increased the maximal respiration rate for palmitate, an effect that was abolished in the presence of catalase (Figure 4A and Supplemental Figure 3B). In contrast, the knockdown of endogenous Nox4 significantly decreased the maximal palmitate respiration rate (Figure 4B). Turning to glucose, the knockdown of Nox4 significantly increased the basal oxidation rate, whereas its overexpression had no significant effect (Figure 4, C and D). In parallel, glycolysis — as assessed by the extracellular acidification rate (ECAR) — was significantly increased by the knockdown of Nox4, while overexpression of Nox4 had no effect (Supplemental Figure 3C). The oxidation rate for pyruvate was unaffected either by knockdown or overexpression of Nox4 (Figure 4, E and F), consistent with a lack of effect on mitochondrial function per se. Overall, these results are similar to the findings in whole hearts.

**Figure 5:**
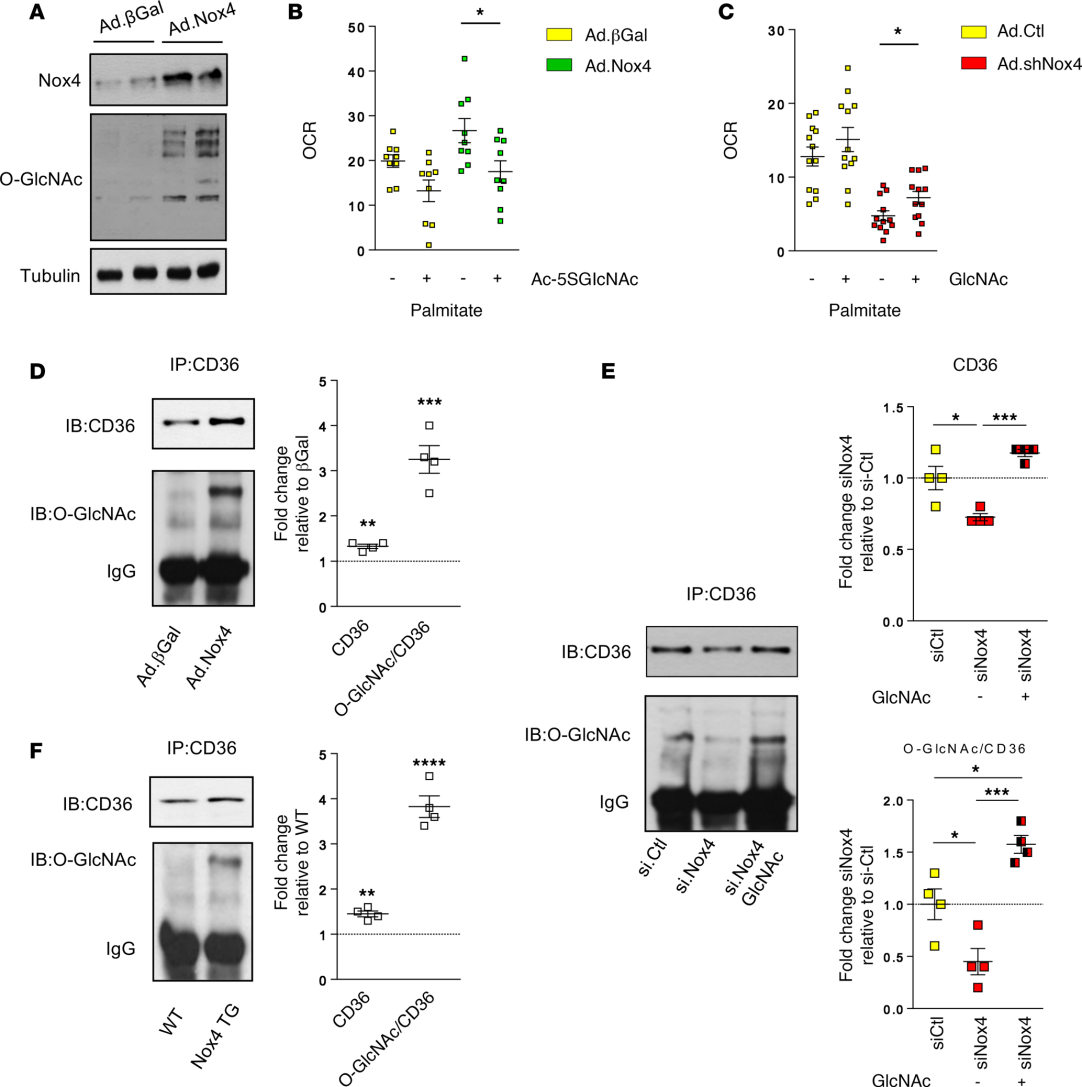
Nox4 modulates fatty acid oxidation via protein O-GlcNAcylation. (**A**) Nox4 overexpression increased protein O-GlcNAcylation in cultured cardiomyocytes. Tubulin was used as a loading control. (**B**) Inhibition of protein O-GlcNAcylation with an HBP inhibitor (Ac-5SGlcNAc, 50 μM) reversed the effect of Nox4 overexpression on oxygen consumption rate (OCR) for palmitate in cardiomyocytes. *P < 0.05, 2-tailed *t* test, *n* = 9. (**C**) In cardiomyocytes in which Nox4 was knocked down, supplementation with GlcNAc (40mM) partially increased the OCR for palmitate. *P < 0.05, 2-tailed *t* test, *n* = 12. (**D**) IP of CD36 in protein lysates (membrane fractions) from cardiomyocytes overexpressing Nox4 or β-Gal control was followed by IB for CD36 and for O-GlcNAcylation (O-GlcNAc). A representative immunoblot is shown to the left. To the right are mean data from 4 independent experiments. **P < 0.01, ***P < 0.001, 2-tailed *t* test. (**E**) IP of CD36 followed by IB for CD36 and O-GlcNAc in cardiomyocytes with siRNA-mediated knockdown of Nox4, with and without supplementation of GlcNAc (100 μM), or in cells treated with a control siRNA. A representative immunoblot is shown to the left. To the right are mean data from 4 independent experiments. *P < 0.05, ***P < 0.001, 1-way ANOVA using Tukey’s multiple comparison test. (**F**) IP of CD36 followed by IB for CD36 and O-GlcNAc in membrane fractions from WT and Nox4 TG hearts. A representative immunoblot is shown to the left. To the right are mean data from 4 independent experiments. **P < 0.01, ****P < 0.0001, 2-tailed *t* test. See also Supplemental Figure 4.

We further assessed potential mechanisms underlying the Nox4-dependent increase in fatty acid oxidation rate. There were no changes in the expression levels of carnitine palmitoyl transferase 1 and 2 (Cpt1b and Cpt2), which are required for the transport of long-chain fatty acids into mitochondria and their subsequent oxidation (Supplemental Figure 4A). In addition, the levels of malonyl CoA, an important allosteric regulator of fatty acid oxidation, were similar in WT and Nox4 TG hearts (Supplemental Figure 4B). The overexpression of Nox4 in cultured cardiomyocytes resulted in a marked increase in O-GlcNAcylated proteins (Figure 5A), similar to the results in Nox4 TG hearts. Since previous studies have linked cardiac O-GlcNAcylation to increases in fatty acid oxidation (21, 22), we tested whether the Nox4-induced increases in O-GlcNAcylation and fatty acid oxidation were causatively linked. Protein O-GlcNAcylation was inhibited in Nox4-overexpressing cardiomyocytes using Ac-5SGlcNAc, a specific competitive inhibitor of Ogt (23) (Supplemental Figure 4C). We observed that the increase in fatty acid oxidation induced by Nox4 overexpression was abolished in the presence of the O-GlcNAcylation inhibitor (Figure 5B). Conversely, we could partially rescue fatty acid oxidation in Nox4-knockdown cardiomyocytes by augmenting HBP activity with *N*-acetyl-D-glucosamine supplementation (Figure 5C).

The augmentation of fatty acid oxidation by O-GlcNAcylation has been suggested to involve the modification of the fatty acid transporter CD36, which is a key regulator of myocardial fatty acid uptake (21). We found that overexpression of Nox4 in cultured cardiomyocytes was accompanied by substantially increased O-GlcNAcylation of CD36, as assessed by IP studies, along with a smaller increase in total CD36 levels (Figure 5D and Supplemental Figure 4D). Conversely, the knockdown of Nox4 resulted in a significant decrease in CD36 O-GlcNAcylation and total CD36 levels (Figure 5E). When cells in which Nox4 had been depleted were treated with *N*-acetyl-D-glucosamine to augment HBP activity, the reduction in CD36 O-GlcNAcylation and total CD36 levels was reversed (Figure 5E). A similar increase in O-GlcNAcylation of CD36 and total CD36 levels to that observed in Nox4-overexpressing cells was also found in Nox4 TG hearts (Figure 5F). Taken together, these results suggest that a putative mechanism by which Nox4 enhances cardiac fatty acid oxidation is as a consequence of increased O-GlcNAcylation of CD36.

### ATF4 mediates the effects of Nox4 on the HBP.

We next wished to investigate the mechanism through which Nox4 increases the activity of the HBP. Given the build-up of proximal glycolytic metabolites, it is possible that Nox4 may be inhibiting forward flux in the glycolytic pathway with a secondary increase in HBP activity. A potential point at which enzyme inhibition may cause such an accumulation of proximal metabolites is GAPDH, which catalyzes the conversion of glyceraldehyde 3-phosphate to 1,3-bisphosphoglycerate (BPG). Interestingly, previous work has shown that GAPDH hyperoxidation inhibits its activity (24). We therefore looked for evidence of GAPDH hyperoxidation in Nox4 TG hearts using an antibody against dimedone-conjugated sulfenate (SOH), where hyperoxidation to SO_2_/SO_3_ results in loss of the dimedone signal. However, no evidence of GAPDH hyperoxidation was found (Supplemental Figure 5A). Similar experiments in cultured cardiomyocytes overexpressing Nox4 also failed to show evidence of GAPDH hyperoxidation (Supplemental Figure 5B). Furthermore, we found no evidence of reduction in GAPDH activity in Nox4 TG compared with WT hearts; in fact, there was a small increase in activity (Supplemental Figure 5C).

**Figure 6:**
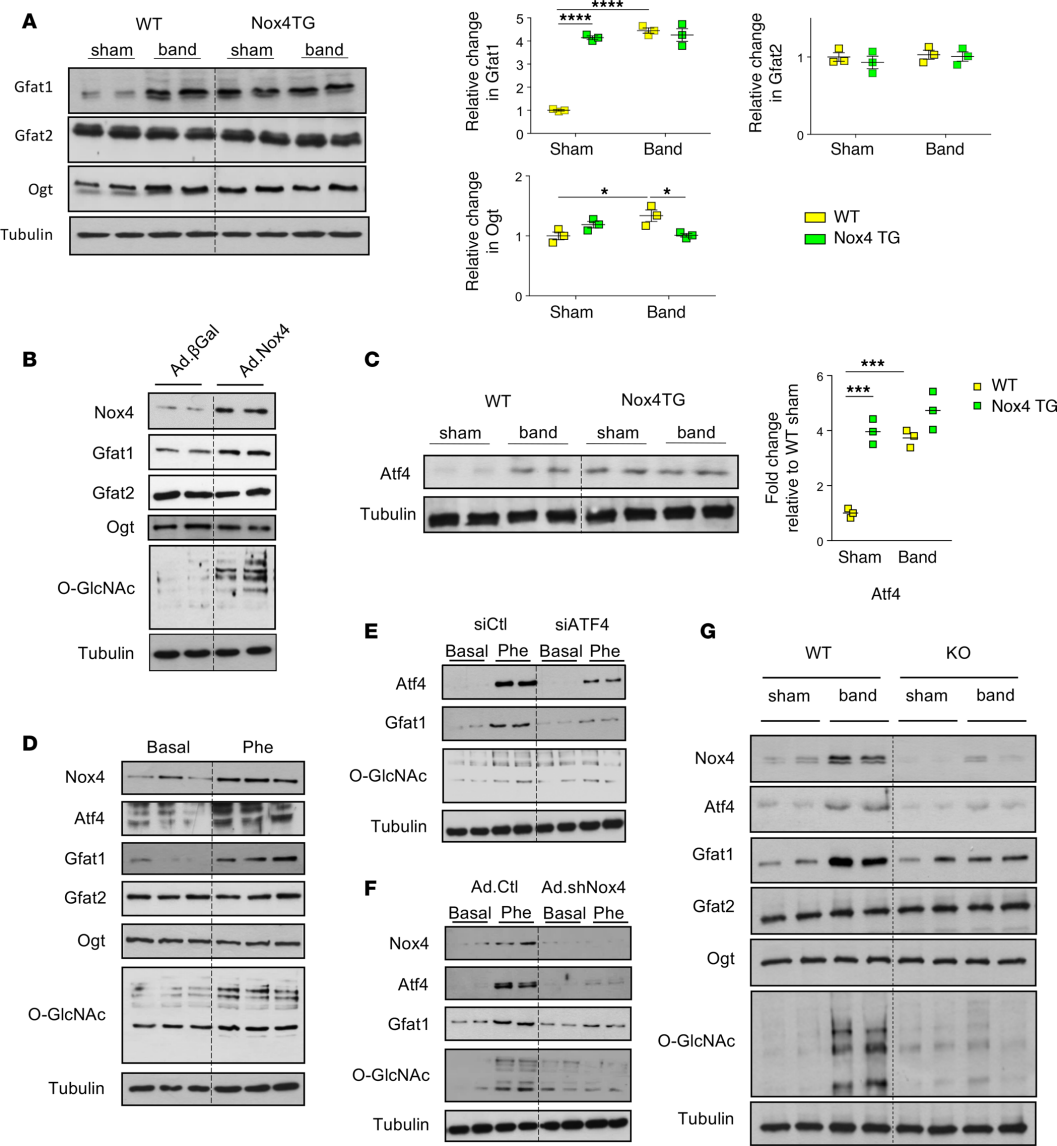
Nox4 regulates Atf4-Gfat1-O-GlcNAc signaling. (**A**) Protein levels of the HBP enzymes Gfat1, Gfat2, and Ogt in WT and Nox4 TG hearts after chronic pressure overload (Band) or control (Sham). Mean data to the right. *P < 0.05, ****P < 0.0001, 2-way ANOVA with Tukey’s post hoc correction, *n* = 3. (**B**) Effect of overexpression of Nox4 or β-Gal in cultured cardiomyocytes on protein levels of HBP enzymes. Mean data are shown in Supplemental Figure 6A. (**C**) Protein levels of Atf4 in WT and Nox4 TG hearts after aortic banding or sham. Mean data are shown to the right. ***P < 0.001, 2-way ANOVA with Tukey’s post hoc correction, *n* = 3. (**D**) Treatment of cardiomyocytes with phenylephrine (10 μM, 24 h) induced an increase in protein levels of Nox4, Atf4, Gfat1, and protein O-GlcNAcylation. (**E**) Effect of silencing Atf4 on phenylephrine-induced changes in Gfat1 and protein O-GlcNAcylation. (**F**) Effect of silencing Nox4 on phenylephrine-induced changes in Atf4, Gfat1, and protein O-GlcNAcylation. (**G**) Protein levels of the HBP enzymes and Atf4 in WT and Nox4-KO hearts after chronic pressure overload (Band) or control (Sham). Mean data for **D**–**G** are shown in Supplemental Figure 6.

We then considered the possibility that Nox4 may more directly influence the HBP. The rate-limiting step for UDP-GlcNAc synthesis is the conversion of fructose 6-phosphate to glucosamine 6-phosphate by glutamine fructose-6-phosphate aminotransferase (Gfat). We found that there was a significant increase in Gfat1 protein level in unstressed Nox4 TG hearts compared with WT hearts, but there was no difference in the levels of the Gfat2 isoform, while Ogt was slightly increased (Figure 6A). Gfat1 and Ogt protein levels increased after banding in the WT group. The overexpression of Nox4 in cultured cardiomyocytes also increased Gfat1 levels without changes in Gfat2 or Ogt (Figure 6B and Supplemental Figure 6A). We recently identified a highly specific molecular stress signaling pathway through which Nox4 upregulates the transcription factor ATF4 (16). In light of a recent report that Gfat1 is a direct target of ATF4 (25), we investigated whether Nox4 may influence protein O-GlcNAcylation in the heart via an ATF4/Gfat1 pathway. Consistent with this possibility, the hearts of Nox4 TG mice demonstrated increased protein levels of ATF4 as compared with WT hearts, while the induction of pressure overload in WT mice (which increases Nox4 levels; Supplemental Figure 1A) also increased ATF4 levels (Figure 6C). In cultured cardiomyocytes, treatment with the hypertrophic agonist phenylephrine significantly increased the levels of Nox4, ATF4, and Gfat1, along with increasing O-GlcNAcylated proteins (Figure 6D and Supplemental Figure 6B). The siRNA-mediated knockdown of ATF4 substantially inhibited the phenylephrine-induced increase in levels of Gfat1 and protein O-GlcNAcylation (Figure 6E and Supplemental Figure 6C). In turn, the knockdown of Nox4 abrogated phenylephrine-induced increases in ATF4 levels and also resulted in an inhibition of the stress-induced increase in Gfat1 and protein O-GlcNAcylation (Figure 6F and Supplemental Figure 6D). Taken together, these data indicate that the Nox4-induced increase in protein O-GlcNAcylation is driven via an ATF4-mediated increase in levels of the rate-limiting enzyme Gfat1.

Finally, we wanted to assess whether endogenous Nox4 mediates similar ATF4/Gfat1 signaling in vivo. We therefore analyzed hearts from Nox4-KO mice and WT littermates subjected to abdominal aortic banding. Whereas WT hearts showed significant increases in the protein levels of Nox4, ATF4, Gfat1, and O-GlcNAcylation after imposition of chronic pressure overload, these changes were substantially inhibited in Nox4-KO hearts (Figure 6G and Supplemental Figure 6E). These results confirm that endogenous Nox4 regulates cardiac ATF4/Gfat1/O-GlcNAcylation signaling in response to chronic pressure overload in WT mice.

### Nox4 TG hearts have preserved cardiac energetics and function.

A decrease in fatty acid oxidation in the chronically stressed or failing heart is suggested to be an adaptive response (5), but here we report a significant increase in fatty acid oxidation in Nox4 TG mouse hearts; however, these animals display a beneficial phenotype in the setting of chronic pressure overload, with less cardiac remodeling and dysfunction (13). We therefore wished to more directly assess the cardiac energetic state in these animals.

**Figure 7:**
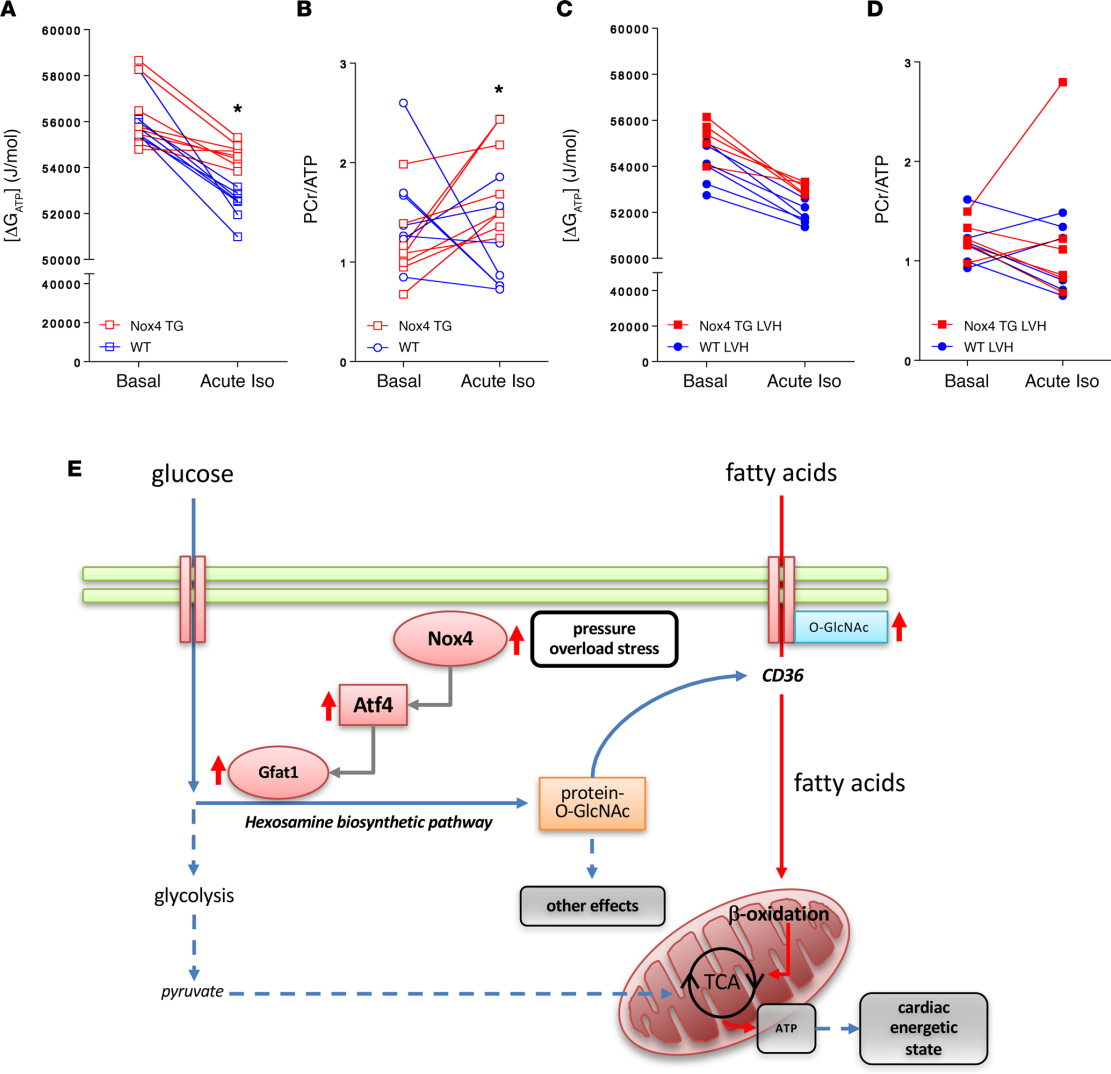
Effects of Nox4 on cardiac energetic state. (**A–D**) Hearts were isolated from Nox4 TG and WT mice under control conditions (**A** and **B**) or after chronic pressure overload (LVH, **C** and **D**) and assessed by ^31^P-NMR spectroscopy. Energetic state was assessed at baseline (Basal) and after acute exposure to isoproterenol (Iso, 50 nM). The Gibbs free energy ([ΔG_ATP_]) and the phosphocreatine/ATP (PCr/ATP) ratio are shown. *P < 0.05 (WT vs. Nox4 TG), *n* = 7–8 per group, 2-way ANOVA with Sidak’s multiple comparison’s test. (**E**) Schematic depicting the effect of increased Nox4 levels on glucose and fatty acid metabolism. Nox4 augments Atf4/Gfat1 signaling to increase HBP activity and protein O-GlcNAcylation. This, in turn, mediates an increase in fatty acid oxidation via an enhancement of CD36 O-GlcNAcylation. This reprogramming of metabolism maintains cardiac energetic state and renders the heart more capable of adaptive remodeling.

We performed cardiac ^31^P-NMR studies in which Nox4 TG and matched WT hearts from unstressed animals or from mice that had been subjected to aortic banding were studied. We also tested the response to super-added acute adrenergic stress in all the groups. Nox4 TG and WT hearts had a similar basal energetic state, as assessed by the Gibbs free energy or the phosphocreatine/ATP (PCr/ATP) ratio (Figure 7, A and B). After an acute challenge with isoproterenol, Nox4 TG hearts had a significantly smaller decrease in Gibbs free energy than WT hearts and a higher PCr/ATP ratio (Figure 7, A and B) despite a similar workload (Supplemental Figure 7A), indicating a better energetic state. Hypertrophied Nox4 TG hearts showed a similar energetic state to WT hypertrophied hearts, both at baseline and after acute isoproterenol challenge (Figure 7, C and D, and Supplemental Figure 7B). These results indicate that Nox4 TG hearts have well-preserved cardiac energetic and functional states.

## Discussion

Alterations in cardiac metabolism may be a crucial component of the mechanisms that enable the heart to successfully adapt to chronically increased workload while it undergoes hypertrophic remodeling (26). However, it remains unclear what changes in metabolic pathways are beneficial in the remodeling heart. The Nox family protein Nox4 is significantly upregulated in the heart in response to hemodynamic overload and promotes adaptive cardiac remodeling (13). In the present study, we demonstrate that Nox4 drives significant alterations in cardiac substrate metabolism, including a relative switch from glucose oxidation to fatty acid oxidation that maintains energetic status, while promoting adaptive remodeling. Nox4 drives an ATF4-mediated increase in activity of the glycolytic branch pathway, the HBP, which facilitates an increase in fatty acid oxidation through O-GlcNAcylation of the fatty acid transporter CD36 (Figure 7E). This reprogramming of cardiac substrate and intermediary metabolism may render the heart more capable of resisting pathological remodeling in the face of chronic hemodynamic stress. Our results, therefore, identify the Nox4/ATF4/HBP axis as a key signaling pathway involved in adaptive metabolic reprogramming in the chronically stressed heart.

Fatty acids are the primary source of energy in the normal well-perfused heart (3). Studies in diverse models show that the fatty acid oxidation rate is generally reduced in pathological LVH, while glycolysis is increased — but without an equivalent increase in the glucose oxidation rate (2–4). The switch from fatty acid to glucose utilization may be part of the reversion to a fetal phenotype characteristic of pathological LVH and is suggested, in principle, to be beneficial because ATP generation from fatty acids is less oxygen efficient than from glucose (3, 27, 28). In addition, the loss of optimal coupling between glycolysis and glucose oxidation may compromise overall myocardial ATP supply and increase the propensity to intracellular acidification. As the heart transitions to overt heart failure, these derangements worsen and coincide with a reduced energetic status, leading to the hypothesis that the failing heart is like an engine out of fuel (29). Current therapies in development for heart failure aim to improve energetic status by enhancing glucose utilization and the coupling between glycolysis and glucose oxidation (5). A striking finding in the current study was that the adaptive remodeling phenotype induced by Nox4 was accompanied by a substantial increase in the fatty acid oxidation rate. Furthermore, there was no evidence of energetic impairment in Nox4 TG hearts during acute neurohumoral stress or chronic hemodynamic stress, or during a combination of acute and chronic stress. These findings indicate that an increase in fatty acid oxidation per se is not necessarily detrimental in the hypertrophied heart but that such an increase may play a central role in the maintenance of energetics in these animals. Our findings are consistent with a number of other recent studies that have reported associations between an increase in fatty acid oxidation and preserved LV function during hemodynamic overload (30–33). It should be noted, however, that the impact of increased fatty acid oxidation may be different in the setting of ischemia or diabetes.

The Nox4-driven increase in fatty acid oxidation is integrally linked to a reprogramming of glucose utilization — in particular, a decrease in glucose oxidation and a marked increase in activity of the HBP as assessed by protein O-GlcNAcylation. We found that the Nox4-dependent enhancement of protein O-GlcNAcylation drives the increase in fatty acid oxidation in cells — findings consistent with previous reports that an increase in HBP activity enhances cardiac fatty acid oxidation (22). Our studies in isolated cardiac mitochondria, as well as the lack of change in levels of mitochondrial fatty acid transporters and malonyl CoA levels, pointed to a nonmitochondrial mechanism as the basis for the increase in fatty acid oxidation. In this regard, an increase in O-GlcNAcylation of the sarcolemmal fatty acid transporter CD36, which is responsible for the majority of cardiac fatty acid uptake (34), was previously suggested to mediate increased fatty acid oxidation in isolated hearts (21, 22). Here, we show that Nox4 drives an increase in O-GlcNAcylation of CD36, both in cells and in hearts in vivo, which interestingly is accompanied by a modest but significant increase in overall levels of CD36. The Nox4-driven increase in HBP activity and protein O-GlcNAcylation, therefore, serves to couple complementary changes in glucose and fatty acid utilization and thereby serves to maintain energetic status.

Very little is known about the mechanisms that regulate the HBP in the heart, for example in the setting of hemodynamic overload (20, 35). The enzyme Gfat is considered to be rate limiting for the production of UDP-GlcNAc, which provides the GlcNAc moiety required for protein O-GlcNAcylation. We found that Gfat1 levels were significantly increased by Nox4, both in cells and in hearts in vivo, without changes in the levels of Gfat2 or Ogt (which catalyzes the transfer of O-GlcNAc from UDP-GlcNAc onto specific proteins). Gfat1 was recently found to be a direct target of ATF4 (25), and here, we were able to demonstrate that the Nox4-dependent upregulation of Gfat1 was dependent on ATF4. Importantly, our studies in Nox4-KO mice — as well as experiments involving the knockdown of Nox4 in cultured cardiomyocytes — confirmed that endogenous Nox4 is a crucial physiological regulator of cardiac ATF4/Gfat1/O-GlcNAcylation signaling during chronic pressure overload. The increase in Nox4 levels during chronic pressure overload is thought to be driven mainly by transcriptional activation, at least in part involving neurohumoral agonists (8, 9). How Nox4 upregulates ATF4 in cardiomyocytes was defined in detail in our recent work. We identified that Nox4 localized in the ER mediates a targeted redox inhibition of protein phosphatase 1 (PP1) in that subcellular compartment, which in turn leads to an increased phosphorylation of the eukaryotic initiation factor eIF2α and an increase in ATF4 translation (16). The mechanism is highly specific to endogenous Nox4 and involves both an increase in Nox4 protein level and its colocalization with its target (PP1) at the ER (16). An ATF4-driven increase in cytoprotective genes is a central mechanism in diverse evolutionarily conserved stress responses (therefore termed the integrated stress response) (36, 37). The present findings indicate that it plays an important role in regulating HBP activity and adaptive metabolic reprogramming downstream of Nox4.

Protein O-GlcNAcylation has attracted increasing attention in recent years, with the recognition that it can modify the function of numerous cellular proteins both under physiological and pathological conditions (20, 35). Previous work found that the inhibition of protein O-GlcNAcylation impaired the response to myocardial ischemia (38), indicating protective effects of O-GlcNAcylation in this context. On the other hand, increased O-GlcNAcylation has also been linked to detrimental effects — for example, in the diabetic heart (35). There is as yet very limited knowledge on the mechanisms that determine which protein targets undergo O-GlcNAc modification in different physiological or pathophysiological settings and how this impacts on overall functional responses. While the protein O-GlcNAcylation of CD36 appears to be an important facet of the metabolic reprogramming observed in the present study, further studies are necessary to define the molecular mechanism underpinning alteration in CD36 function by O-GlcNAcylation, as well as the precise contribution to Nox4-mediated protection during chronic pressure overload. It is likely that the altered O-GlcNAcylation of other proteins may also contribute to the overall adaptive remodeling phenotype induced by Nox4. A notable feature of the metabolic reprogramming induced by Nox4 was that glucose oxidation was decreased, yet glucose uptake was unaltered. The quantification of glycolytic intermediates demonstrated a relative increase in proximal compared with distal intermediates in Nox4 TG hearts. These findings may, at least in part, reflect an increase in flux through the HBP. We speculate that, in addition, there may be an increased flux of glucose into other glycolytic branch pathways that feed intermediary metabolism (such as the pentose phosphate pathway), contributing to the overall beneficial effects of increased Nox4 levels in the heart.

The identification of mechanisms that enable the heart to successfully adapt to a chronically increased workload is central to efforts to develop new therapeutic approaches to prevent and treat chronic heart failure. In this study, we identify a coordinated reprogramming of cardiac fatty acid and glucose metabolism that is driven by Nox4/HBP/O-GlcNAc signaling as an adaptive pathway in the hemodynamically overloaded heart. Our findings indicate that an increase in fatty acid oxidation in this context may be beneficial when it occurs as part of coordinated alterations in glucose and intermediary metabolism, perhaps coupling the maintenance of energetic status to optimal partitioning of glucose utilization in the remodeling heart.

## Methods

### Animals.

Cardiomyocyte-targeted Nox4 TG mice with a C57BL/6 background and Nox4-KO mice were generated as previously described (13). Aortic constriction was performed by suprarenal abdominal aortic banding (13).

### Proteomics.

Two-dimensional DIGE (2D-DIGE) was performed on LV tissue samples labeled with fluorescent cyanine dyes, as previously described (39). Differentially expressed protein spots were excised, digested with trypsin, and analyzed by liquid chromatography tandem MS (LC-MS/MS) on an LTQ Orbitap XL mass spectrometer (Thermo Fisher Scientific) (39). Spectra were searched against a mouse database (SwissProt) using the SEQUEST algorithm (SRF v5) and analyzed by Scaffold software (v2.1.03). The enriched GO biological functions were analyzed using the Reactome database (http://www.reactome.org/). Detailed methods are described in the Supplemental Methods.

### Reverse transcription PCR (RT-PCR), immunoblotting, and immunoprecipitation studies.

Real-time PCR was performed using Sybr Green (Applied Biosystems) and the ΔΔCt method. IB was performed using standard methods, with quantification by enhanced chemiluminescence and densitometry. We used a CD36 antibody (Abcam) coupled to protein A/G agarose for IP. Detailed methods including primer sequences and antibody sources are described in the Supplemental Methods.

### Isolated heart substrate utilization.

Myocardial substrate utilization was quantified in isolated ejecting heart preparations perfused with a mixture of substrates (including glucose and palmitate), as previously described (18). The use of ^14^C-labeled glucose or palmitate and ^3^H-labeled glucose enabled the assessment of glucose or fatty acid oxidation, glucose uptake, and glycolytic flux (Figure 2, A–D). Metabolic rates were normalized by the dry heart weight. Full details are provided in the Supplemental Methods.

### Measurement of glycolytic metabolites by LC-MS.

Metabolites were isolated from flash-frozen hearts using a modified Bligh-Dyer methanol/chloroform method (40). Compounds were separated by liquid-chromatography using a Waters Acquity Ultra Performance Liquid Chromatography unit (Waters Ltd.) and analyzed in negative and positive ion mode in a Sciex 5500 triple quadrupole mass spectrometer (AB Sciex). Full details are provided in the Supplemental Methods.

### Cells and transfections.

Neonatal rat cardiomyocytes were isolated and cultured using standard methods (13). Adenoviral vectors for the overexpression of Nox4 (Ad.Nox4), knockdown of Nox4 with a short hairpin sequence (Ad.shNox4), and respective controls (Ad.βGal, Ad.Ctl), as well as siRNA sequences for the knockdown of ATF4, were previously described (16). Full details are provided in the Supplemental Methods.

### Extracellular flux assays.

Neonatal rat cardiomyocytes were isolated and cultured onto gelatin-coated Seahorse XF^e^24 well-plates. Oxygen consumption rates (OCR) and ECAR were measured according to the manufacturer’s protocol. Full details are provided in the Supplemental Methods.

### ^31^P NMR spectroscopy in isolated hearts.

Hearts were perfused in isovolumic Langendorff mode with modified Krebs-Henseleit buffer containing glucose and octanoate. ^31^P NMR spectroscopy was performed using a Bruker Avance III 400 MHz spectrometer (41). Quantification of Pi, PCr, and β-ATP (mM) and the calculation of intracellular pH and ΔG_ATP_ were performed using established methods (42, 43). Full details are provided in the Supplemental Methods.

### Other biochemical assays.

The respiration of isolated mitochondria was measured using a high-resolution respirometer (OROBOROS Oxygraph-2K). Cardiac mitochondria were isolated (44) and OCR measured for multiple substrates. PDH enzymatic activity was measured in mitochondrial fractions using a commercially available dipstick assay (ab109882, Abcam) and normalized to the relative amount of PDH E1α protein. GAPDH activity was measured in heart tissue lysate using a commercially available colorimetric assay (ab204732, Abcam). Detailed protocols are described in the Supplemental Methods.

### Statistics.

Data are shown as the mean ± SEM. Differences in means were compared by unpaired, 2-tailed Student’s *t* tests, 1-way ANOVA or 2-way ANOVA, followed by a post-hoc test for multiple comparisons, as appropriate. Analyses were performed using GraphPad Prism (v6.0). *P* < 0.05 was considered significant.

### Study approval.

All procedures were performed in accordance with the Guidance on the Operation of the Animals (Scientific Procedures) Act, 1986 (United Kingdom), and/or EU Directive 2010/63 on the use of animals for scientific purposes and were approved by ethical review panels at King’s College London and the Arctic University of Norway.

## Author contributions

AAN, AZ, ADH, CXCS, MZ, MB, XY, JAW, and TRE performed experiments and analyzed data. EA, JLG, and MM designed and supervised experiments. ACB developed the Nox4 TG mouse model. KS developed the Nox4-KO mice. AAN, AZ, ADH, and AMS wrote the paper. EDA provided critical intellectual input. AMS conceived the study and designed and supervised experiments.

## Supplementary Material

Supplemental data

## Figures and Tables

**Table 1:**
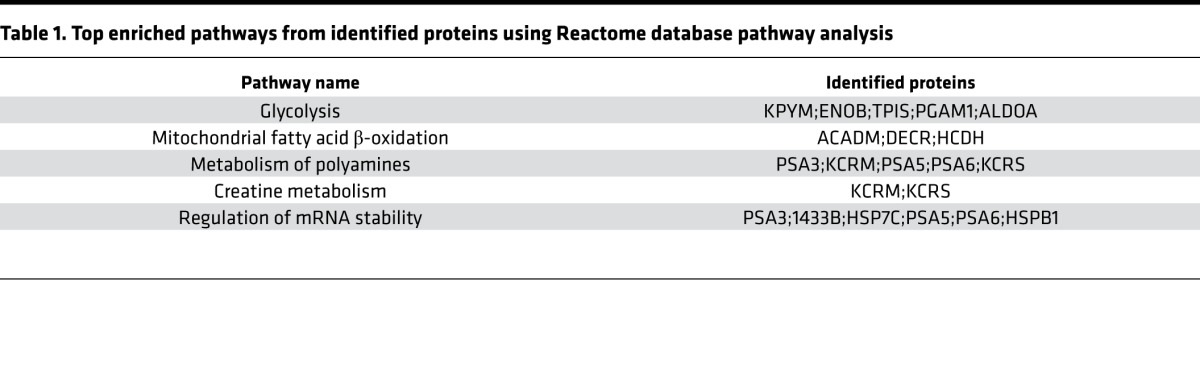
Top enriched pathways from identified proteins using Reactome database pathway analysis

## Version 1 12/21/2017

Electronic publication
