# Supplementary material for: Nox4 reprograms cardiac substrate metabolism via protein O-GlcNAcylation to enhance stress adaptation
Source: JCI Insight. 2017 Dec 21;2(24):e96184. doi: 10.1172/jci.insight.96184 (PMC5752273; doi:10.1172/jci.insight.96184)
Supplement: Supplemental data [file jciinsight-2-96184-s001.pdf]

SUPPLEMENT

Nox4 reprograms cardiac substrate metabolism via protein O-GlcNAcylation to enhance stress adaptation

Adam A. Nabeebaccus, Anna Zoccarato, Anne D. Hafstad, Celio X.C. Santos, Ellen Aasum, Alison C. Brewer, Min Zhang, Matteo Beretta, Xiaoke Yin, James A. West, Katrin Schröder, Julian L. Griffin, Thomas R. Eykyn, E. Dale Abel, Manuel Mayr, Ajay M. Shah.

Supplemental methods

Chemicals. All chemicals were purchased from Sigma Aldrich or Calbiochem, unless otherwise indicated, and were of analytical or higher HPLC purity grade.

Animal studies. Procedures were performed in accordance with the UK Home Office 'Guidance on the Operation of the Animals' (Scientific Procedures) Act, 1986 or the Norwegian National Animal Research Authority (NARA) guidelines and the EU Directive 2010/63 on the use of animals for scientific purposes. Cardiomyocyte-targeted *Nox4*-transgenic mice on a C57Bl/6 background and Nox4 knockout mice were described previously (1). Supra-renal abdominal aortic banding was used to induce pressure-overload left ventricular (LV) hypertrophy which is initially associated with compensated cardiac function and then gradually progresses to significant contractile impairment (2). Sham surgery was identical apart from placement of the band.

Proteomics. LV heart tissue was analyzed by Difference in-gel electrophoresis (DIGE) as described previously (3). Frozen tissue was homogenized in lysis buffer (9.5 M Urea, 2 % w/v CHAPS, 1 % w/v DTT, 2 % v/v Pharmalyte pH 3-10) containing protease inhibitors. Insoluble membrane components (including myofilaments) were removed by centrifugation and the supernatant was analyzed. Samples were processed using the ReadyPrep 2-D Cleanup Kit (BioRad) and re-dissolved in DIGE lysis buffer (8 M Urea, 4 % w/v CHAPS, 30 mM Tris-HCl, pH = 8.5). Equal amounts of samples were labeled with fluorescent cyanine dyes, Cy3 or Cy5, and Cy2 was used to label a pooled internal standard (Amersham CyDye DIGE Fluor Minimal Labeling Kit, GE Healthcare). Lysine (10 mM) was added to stop the labeling reaction. The same amount of Cy2-, Cy3- and Cy5-labeled samples were pooled together and 2X DIGE buffer (8 M urea, 4% w/v CHAPS, 2% w/v DTT, 2% v/v Pharmalyte pH 3-10) was added to equal volumes of total protein lysate. Rehydration buffer (8 M Urea, 0.5% w/v CHAPS, 0.2% w/v DTT, 0.2% v/v Pharmalyte pH 3-10) was added to make a final volume of 450 µL and the Immobiline™ DryStrips (pH 3-10 NL, 18 cm, GE Healthcare) were rehydrated overnight at room temperature. Isoelectric focusing (IEF) was performed using the IPGphor IEF system (GE Healthcare) at 20°C with the following protocol: 150 V for 2 h, 300 V for 2 h, 600 V for 2 h, 1500 V for 8 h, 1500 V - 8000 V in 30 min, then 8000 V for 2 h. After the IEF had finished, the IPG strips were left in equilibration buffer (50 mM Tris-HCl pH 8.8, 6 M Urea, 2% w/v SDS, 30% w/v Glycerol, and bromophenol blue) with 10 mg/mL DTT for 15 min on a shaker, followed by 15 min incubation with equilibration buffer with 48 mg/mL iodoacetamide. The strips were briefly washed with water and loaded onto 12% polyacrylamide gels without a stacking gel. Gels were run in an Ettan DALTsix electrophoresis system (GE Healthcare) at 10°C with the following protocol: 600V, 400mA, 2W/gel for 15 min, 2.5W/gel for 30 min and

16W/gel until the blue dye front reached the bottom of the gels. After electrophoresis, gels were scanned using a fluorescent scanner (Ettan DIGE Imager, GE Healthcare) to detect Cy2, Cy3 and Cy5 signal. Next, the gels were fixed (40% methanol, 10% acetic acid in 18MΩ water) and silver stained (PlusOne silver staining kit for Protein, GE healthcare). Silver stained images were scanned on a flatbed scanner (GS-800, BioRad) for documentation. The fluorescent images were analyzed by automated software (DeCyder 2D version 7.0, GE Healthcare). Protein spots with >1.2 fold change and $p<0.05$ were excised using a OneTouch 2D gel spot picker (1.5 mm P2D1.5, The Gel Company) to yield small gel plugs that were digested with trypsin in an Investigator ProGest Digester (DIGILAB). Digested peptides were lyophilized and reconstituted in 2% acetonitrile, 0.05% TFA for separation by nano-flow liquid chromatography (Dionex UltiMate 3000) on a reverse-phase column (PepMan100, C18, 15cm x 75um, Thermo Fisher Scientific) connected to a linear ion trap mass spectrometer (LTQ XL, Thermo Fisher Scientific) for tandem mass spectrometry (MS/MS). Full MS scan mode was performed over an m/z range of 450-1600. MS/MS was performed on the six most abundant ions for each full MS scan using collisional induced dissociation (CID) with dynamic exclusion enabled. Spectra were searched against a mouse database (SwissProt database version 14.6, 16022 protein entries) using the SEQUEST algorithm (SRF v.5). The following parameters were used: precursor mass tolerance=1.5 Da, fragment mass tolerance=0.8 Da, carbamidomethylation of cysteine was used as fixed modification and oxidation of methionine as variable modification. Trypsin was used and 2 missed cleavages were allowed. Peptide results were analyzed by Scaffold software (v. 2.1.03). Enriched GO biological function was analyzed using an online database (Reactome; http://www.reactome.org/).

Real time-RT PCR. RNA was extracted using the SV Total RNA Isolation System (Promega). Real-time PCR was performed using SYBR®Green PCR Master Mix Technology (Applied Biosystems). β-actin was used for normalization unless specified otherwise. The relative fold change was calculated based on the ΔΔCt method. Primer sequences were obtained from Sigma-Aldrich: β-actin F: 5′ –CGTGAAAGATGAGACCCAGATCA-3′ and R: 5′-TGGTACGACCAGAGGCATACAG-3′. Eno3 F: 5′-GTGTCCCTGGCTGTCTGC-3′ and R: 5′-CCGCCGTTGATCACATTA-3′. Acadl F: 5′-GCTTATGAATGTGTGCAACTCC-3′ and R:5′-ACCGTAGATCGGCTGCAC-3′. Acadm F: 5′-AGTACCCTGTGGAGAAGCTGAT-3′ and R:5′-TCAATGTGCTCACGAGCTATG-3′. Hadh F:5′- CTGGCCTCCACTTTTTCAAC-3′ and R:5′-CAAAAGTCCACCAGAGATTCAA-3′. Aldoa F:5′-TGCCAGTATGTTACTGAGAAGGTC-3′ and R:5′-CATGTTGGGCTTCAGCAA-3′. Pkm1 F:5′- TATTCGAGGAACTCCGCCG-3′ and R:5′-TGAGCACTCCTGCCAGACTTG-3′. Pgam1 F:5′- TTCTACAGCAACATCAGCAAGGA-3′ and R:5′-GGCAGTGCTCTGGCAATAGTG-3′. Acaa2 F:5′- GGACATGGATTTGATAGACGTG-3′ and R:5′-GCCTCCACTCACATTGGTTT-3′. Tpi F:5′- AAACCAAGGTCATCGCAGATA-3′ and R:5′-CCCGGAGCTTCTCGTGTA-3′. Cpt1b F:5′-ATCACTGCCCAAGCTTCCT-3′ and R:5′-CCGCACAGAATCCAAGTACC-3′ Cpt2 f:5′-TTCTGCAGTCCGGTTTCTG-3′ and 5′-TTTTGGAAGGGTTCAAGTGG-3′.

Immunoblotting and immunoprecipitation. Heart tissue or cells were lysed in lysis buffer (25 mM Tris, 150 mM NaCl, 2 mM EGTA, 5 mM EDTA, 0.5% NP-40 to pH 7.4) containing a protease and phosphatase inhibitor cocktail (Sigma-Aldrich). In the case of cell lysates used for Nox4 and ATF4 immunoblotting, the lysis buffer also contained a proteasome inhibitor (Mg132, 2 µg/ml). Protein content was estimated by a Bradford Assay and equal amounts of lysates or protein extracts were loaded onto SDS polyacrylamide gels, and then transferred

to nitrocellulose membranes. The primary antibody sources were: Nox4 (4); ATF4 (Santa Cruz C-20, SC-200); Akt (Cell Signaling #4691); Phospho-Akt (Cell Signaling #4051); tubulin and GAPDH (Sigma Aldrich G8795); CD36 (Abcam ab133625); O-GlcNAc mouse monoclonal, RL2 (Abcam ab2739); Ogt (Cell Signaling 5368S); Gfat1 (Abcam ab125069); Gfat2 (Abcam ab190966); Tpi (Abcam ab28760); Pkm (Proteintech 15821-1-AP); Eno3 (ProteinTech 16421-1-AP); Acaa2 (ProteinTech 11111-1-AP); Aldoa (Sigma-Aldrich AV48130); Pgam1 (Abcam ab96622); Acadl (Abcam ab82853); Acadm (Abcam ab108192);Hadh (Abcam ab107260); PDH E1α (Abcam ab155096); PDH E1α (pS293) (Abcam ab92696); VDAC (Abcam ab14734)The antibody against dimedone-conjugated sulfenate (SOH) was a kind gift from Prof. Phil Eaton (King's College London, London, UK). After incubating with horseradish peroxidase (HRP)-linked secondary antibodies, the blots were revealed by chemiluminescence. Densitometric analyses were performed using Image J (NIH, USA).

The immunoprecipitation (IP) protocol was adapted from Laczy et al. (5). Equal aliquots of heart tissue or cell lysates were reserved as input or further processed to obtain membrane fractions. The final membrane pellet was resuspended in lysis buffer, protein concentration normalized to 1 µg/µl and immunoprecipitation was performed using 250 µg of lysate incubated with the CD36 antibody or with non-specific IgG as a negative control. This mixture (200 µL) was gently mixed at 4°C for 1 h, after which 25 µL of bead slurry (Protein A/G PLUS-Agarose; Santa Cruz sc-2003) was added and the mixture further incubated for 16 hours. The beads were spun down (1000 g), washed 7X with RIPA buffer without detergents, and samples were processed for immunoblotting with antibodies against CD36 and O-GlcNAc modification.

Isolated heart substrate utilization. Myocardial substrate utilization was quantified in isolated working hearts using a closed recirculating perfusion system as previously described (6). Hearts were perfused with Krebs-Henseleit (KH) buffer containing glucose (10 mM), palmitate (0.4 mM), lactate (1.5 mM), pyruvate (0.2 mM), glutamine (0.5 mM), insulin (100 IU/mL), and isoproterenol (0.5 nM) The preload pressure was set to 10 mmHg and the afterload pressure to 50 mmHg. Substrate fluxes were measured with one ^{14}C-labeled substrate (either D[U-^{14}C-glucose] (Perkin Elmer) or 1-^{14}C-palmitate (Amersham GE Healthcare)) and one ^{3}H-labeled substrate (either D[2-^{3}H]glucose or D[5-^{3}H]glucose (Perkin Elmer)), using the readouts shown in Figure 2A-D. Glycolytic flux (7, 8) and glucose uptake (9) were determined by measuring the accumulation of $^{3}H_2O$ released into the buffer during the perfusion protocol. Vacuum sublimation was used to extract H_2O from the buffer samples at each time point. Palmitate or glucose oxidation were measured by trapping of $^{14}CO_2$ from the buffer samples collected at each time-point. Buffer samples were injected into a sealed tube with 0.75 mL of sulphuric acid (4.5 M) in order to drive the formation of CO_2 from bicarbonate, which was trapped onto a filter paper with hyamine hydroxide. Simultaneously, the overflow of gas escaping the system was bubbled through hyamine hydroxide in order to trap the rest of the CO_2 formed in the TCA cycle. Vials with the collected $^{3}H_2O$ and $^{14}CO_2$ from each time-point were added to 4 mL of scintillation fluid (Ultima Gold, Perking Elmer) and counted in a scintillator counter. For every experiment, 100 µL aliquots were collected and the amount of radioactivity was counted in order to estimate the specific activity (DPM per µmol palmitate or glucose). Metabolic rates were calculated as the accumulated values of either $^{14}CO_2$ or $^{3}H_2O$ production at each time-point, expressed as turnover of either glucose or palmitate (µmol/minute) and normalized to the mass of dry heart.

LC-MS for quantification of glycolytic metabolites. Metabolites were extracted from 25mg heart tissue using a methanol:chloroform (2/1, v/v) method (10). Samples were homogenized using a Tissue Lyser (Qiagen) for 10 min at 25 Hz. 200 µL of H_2O and 200 µL of $CHCl_3$ were added and after vigorous mixing the samples were centrifuged at 13200 rpm for 25 min. The aqueous fraction (top layer) and organic fraction (bottom layer) were separated and aliquoted into separate tubes. This extraction was repeated once again and the resulting layers were dried under nitrogen. The dried protein pellet was weighed. Samples were reconstituted in 200 µL 7:3 acetonitrile:water (CH_3CN:H_2O) containing 10 µM ^{13}C and ^{15}N labelled glutamate (Cambridge Isotope Laboratories, Tewkesbury, MA, USA), used as internal standards. Samples were transferred to 300 µL glass vials with inserts (Chromacol, Welwyn Garden City, UK)

Compounds were analyzed using a Sciex 5500 triple quadrupole mass spectrometer (AB Sciex, Redwood City, CA, USA) interfaced with a Waters Acquity Ultra Performance Liquid Chromatography unit (Atlas Park, Manchester, Lancs., UK). Samples and standards were chromatographically separated using a BEH amide column (100 mm × 2.1 mm, 1.7 µm; Waters Ltd, Atlas Park, Manchester, Lancs., UK). Mobile phases for the LC gradient consisted of 20 mM ammonium acetate adjusted to pH 9.5 using ammonium hydroxide (29%) (A) and acetonitrile (B). The chromatography gradient was: a starting composition of 10% A, which was increased to 60% A over 4.5 min with re-equilibration for the final 3 min to give a total run time of 7 min. The flow rate was 600 µL/min and the column was kept at ambient temperature. The MS parameters were: capillary voltage -4500 V in negative ion mode and 5500 V in positive ion mode, source temperature 650 °C, gases 1 and 2 both set to 60 psi, curtain gas set to 35 psi, collision gas set to high and the dwell time for all analytes 10 ms.

Data were processed using the quantitation wizard tool of Analyst (AB Sciex, Warrington, UK) where chromatographic peak areas were integrated using a proprietary algorithm and normalized to the internal peak area. Data were further normalized to dry protein pellet weight and collated using Excel (Microsoft Corporation, Redmond, WA, USA).

Cells and transfections. Primary neonatal rat cardiomyocyte cultures were prepared using standard methods (11). Cells were transduced with Adenoviral vectors for the overexpression of Nox4 (Ad.Nox4) or control β- galactosidase (Ad.βGal) at a multiplicity of infection (MOI) of 1 (11). For the knockdown of Nox4, an adenoviral vector expressing a short hairpin sequence targeted against Nox4 (Ad.shNox4) was used, with a vector expressing a sequence targeted against GFP as control (Ad.Ctl) at MOI 20 (12), and cells were studied 72 h later. Short interfering RNAs (siRNAs) were purchased from Qiagen and were transfected using Hyperfect reagent (Qiagen) as described previously (11). siRNA sequences were: rat ATF4(1): F: 5`GCCUAGGUCUCUUAGAUGATT-3`, R: 5`-UCAUCUAAGAGACCUAGGCTT-3`.

^{31}P NMR spectroscopy in isolated hearts. Hearts were quickly excised, canulated and perfused in the isovolumic Langendorff mode, using an oxygenated (95 % O2/5% CO2) modified phosphate-free KHB (118 mM NaCl, 5.9 mM KCl, 1.16 mM $MgSO_4$, 25 mM $NaHCO_3$, 0.48 mM EDTA, 10 mM glucose, 2.2 mM $CaCl_2$, 0.4 mM sodium octanoate) at 37 °C. Perfusion pressure was set to 70 mmHg. The end-diastolic pressure was set to 8 mmHg with a left ventricular balloon. LV pressure was recorded using a Power Lab acquisition module and analyzed using Lab Chart 7.0 (ADInstruments). After 15 min of perfusion, the heart was secured inside a watertight glass tube filled with modified KHB. A sealed capillary tube

containing ethylene glycol inside the glass tube was used for temperature calibration. The apparatus was inserted into the cavity of a Bruker Avance III 400 MHz wide-bore spectrometer coupled with triple axis gradients, a microimaging probe and exchangeable RF coil inserts. Shimming was carried out on the ^{1}H signal of water prior to the ^{31}P acquisition. Fully relaxed ^{31}P spectra were acquired with an experiment duration of 16 min, 256 scans, repetition time of 3.85 s and a 60° flip angle. ^{31}P spectra were acquired for each heart at baseline and during perfusion of isoproterenol (50 nM) as an acute inotropic challenge. Quantification of ^{31}P concentrations of Pi, PCr and β-ATP (mM) was carried out by measuring peak integrals of the relevant metabolites referenced to the peak integral of a 20 mL standard of trisodium trimetaphosphate (200 mM), and scaled to heart mass assuming an intracellular volume of 0.44 mL/gram wet weight. Intracellular pH was calculated from the chemical shift difference (Δppm) between the PCr peak and the P_i peak using the formula: pH = 6.694 + log10 ((Δppm - 3.121)/(5.498 - Δppm)). ΔG_{ATP} was calculated using the following formula: $\Delta G_0 + RT\,ln([ADP][P_i]/[ATP])$ (13). [ADP] was derived from the creatine kinase equilibrium equation: $[ADP] = [ATP][Cr]/[PCr][H^+]Keq$. Rearranging yields an expression for the Gibbs free energy $\Delta G_{ATP} = \Delta G_0 + RT\,ln([Cr][P_i]/[PCr][H^+]Keq)$. Mean creatine concentrations (mM) (assuming an intracellular volume fraction of 0.44 mL/mg tissue) were previously measured from ^{1}H-NMR heart extracts from wild-type and Nox4 TG hearts (WT (sham) 16.1 ± 1.9 mM, Nox4 TG (sham) 12.6 ± 1.0 mM, WT (band) 19.2 ± 1.5 mM and Nox4 TG (band) 13.0 ± 0.7 mM). The value for Keq was taken to be 1.66 x 109 $mol^{-1}L^{-1}$ and ΔG0 = -30.5 $kJmol^{-1}$ (13, 14).

Isolated heart mitochondria studies. Cardiac mitochondria were isolated according to the method described by Palmer (15). Briefly, ventricles were homogenized and trypsinized (5

mg/mL) in isolation buffer (250 mM sucrose, 0.5 mM EDTA, 10 mM Tris; pH 7.4). After differential centrifugation, the mitochondrial pellets were suspended in buffer containing 0.5 mM EGTA, 3mM $MgCl_2$, 60 mM K-lactobionate, 20 mM taurine, 10 mM KH_2PO_4, 20 mM HEPES, 110 mM sucrose, 1 g/L BSA, 280 U/mL catalase, 20 mM histidine, 20 µM vitamin E succinate, 3 mM glutathione, 1 µM leupeptin, 2 mM glutamate, 2 mM malate, and 2 mM ATP, pH 7.1. Mitochondria were kept on ice until study.

The oxygen consumption rate (OCR) was measured using a high-resolution respirometer (OROBOROS Oxygraph-2k, Innsbruck, Austria). Before each experiment, the oxygen concentration in the medium was equilibrated for 15-30 min with air in the respirometer chambers at 37°C until a stable signal was obtained. The isolated mitochondria were then added to the chamber (2 mL volume) containing mitochondrial respiration medium (0.5 mM EGTA, 3 mM $MgCl_2$, 60 mM K-lactobionate, 20 mM taurine, 10 mM KH_2PO_4, 20 mM HEPES, 110 mM sucrose, 1 g/L BSA, pH 7.1) and closed by inserting stoppers. All chemicals were injected using Hamilton syringes. Respiration (Leak state) was measured in the presence of two different respiratory substrates: A (2 mM malate, 5mM L-carnitine and 25 µM palmitoyl CoA) or B (2 mM malate and 5 mM pyruvate). ADP (60 µM) was added to achieve mitochondrial oxidative phosphorylation (OXPHOS) capacity. $Leak_O$ state respiration was recorded after depletion of ADP and addition of oligomycin (4 µg/mL).

PDH enzyme activity. Mitochondrial particulate fractions were prepared from freshly isolated whole heart tissue. PDH enzyme activity was performed using a PDH enzyme activity assay kit (Abcam ab109882) according to the manufacturer’s instructions.

Extracellular flux analyses Cells (100,000 cells/well) were plated on Seahorse XFe24 culture plates pre-coated with 1% gelatin in DMEM-based plating medium (Sigma, #D6546), supplemented with 17% Medium 199 (Sigma, #M2154), 10% horse serum, 5% non-heat inactivated FBS, 1% penicillin/streptomycin, 2 mM glutamine, and 1% non-essential amino acids, and incubated at 37 °C for 12 h. The following day, plating media was switched to DMEM maintenance medium (Sigma, #D5671), supplemented with 20% Medium 199, 1% penicillin/streptomycin and 2 mM glutamine. After 4 h, cells were infected with adenoviruses to manipulate Nox4 levels or control adenoviruses. On the day of the experiment, the maintenance medium was changed for unbuffered DMEM (D5030, Sigma-Aldrich) supplemented with 10 mM Glucose or 2 mM Sodium pyruvate, pH 7.4, at 37 °C, 1 h before assay. For fatty acid oxidation (FAO) experiments, the plating medium was changed with FAO medium (NaCl 111 mM, KCl 4.5 mM, $MgSO_4$ 2mM, Na_2HPO_4 1.2mM, 0.5 mM Glucose, 0.5 mM Carnitine, 5 mM HEPES) 1 h prior to assay, and BSA-conjugated palmitate 0.2 mM or vehicle control was added and the assay undertaken according to the manufacturer's protocols for mitochondrial stress tests. Sodium palmitate (1 mM) (Sigma, P9767) was conjugated to ultrafatty acid-free BSA (0.17 mM) (Roche, 0311740501) following the Seahorse Bioscience protocol (www.seahorsebio.com). Etomoxir (80 µM) was used to confirm that oxygen consumption was due to oxidation of palmitate. Basal oxygen consumption rate (OCR), ATP-dependent, FCCP-stimulated and extra-mitochondrial OCR were determined by the sequential administration of oligomycin (1 µM), FCCP (2 µM), and rotenone and antimycin A (2 and 4 µM respectively). Basal and FCCP-stimulated OCR was adjusted for extra-mitochondrial OCR. For glycolysis, on the day of the experiment the maintenance medium was changed for unbuffered DMEM (D5030, Sigma-Aldrich) pH 7.4, at 37 °C, 1 h before assay and the extracellular acidification rate (ECAR) was determined at

baseline and in response to 10 mM Glucose. For *N*-acetyl-D-glucosamine experiments, myocytes were treated with 40 mM *N*-acetyl-D-glucosamine (A8625, Sigma-Aldrich) overnight and the mitochondrial stress tests were performed in FAO medium supplemented with 40 mM *N*-acetyl-D-glucosamine. For experiments with the OGT inhibitor, cardiomyocytes were treated for 24 h with 50 µM Acetyl-5SGlcNAc (kindly provided by Prof David Vocadlo, Simon Fraser University, Canada), and the mitochondrial stress tests were performed in FAO medium supplemented with 50 µM Acetyl-5SGlcNAc.
Catalase−polyethylene glycol (PEG-catalase, Sigma C4963) was used at a final concentration of 500 U/mL. Myocytes were treated with PEG-catalase for 72 h and the mitochondrial stress tests were performed in FAO medium supplemented with PEG-catalase. Data were normalized by the number of cells seeded.

Statistics. Statistical analyses were performed using GraphPad Prism (v6.0). Data are reported as mean ± SEM. Differences in means were compared by either an unpaired Student's t-test or where appropriate, one-way ANOVA or two-way ANOVA followed by a post-hoc test for multiple comparisons. $P<0.05$ was considered significant.

References

1. Zhang M, et al. NADPH oxidase-4 mediates protection against chronic load-induced stress in mouse hearts by enhancing angiogenesis. *Proc Nat Acad Sci USA.* 2010;107(42):18121-6.
2. Byrne JA, Grieve DJ, Bendall JK, Li JM, Gove C, Lambeth JD, Cave AC, Shah AM. Contrasting roles of NADPH oxidase isoforms in pressure-overload versus angiotensin II-induced cardiac hypertrophy. *Circ Res.* 2003;93(9):802-5.
3. Yin X, et al. Effects of perhexiline-induced fuel switch on the cardiac proteome and metabolome. *J Mol Cell Cardiol.* 2013;55:27-30.
4. Anilkumar N, Weber R, Zhang M, Brewer A, Shah AM. Nox4 and Nox2 NADPH oxidases mediate distinct cellular redox signaling responses to agonist stimulation. *Arterioscler Thromb Vasc Biol.* 2008;28(7):1347-54.
5. Laczy B, Fulop N, Onay-Besikci A, Des Rosiers C, Chatham JC. Acute regulation of cardiac metabolism by the hexosamine biosynthesis pathway and protein O-GlcNAcylation. *PloS one.* 2011;6(4):e18417.

6. Aasum E, Hafstad AD, Severson DL, Larsen TS. Age-dependent changes in metabolism, contractile function, and ischemic sensitivity in hearts from db/db mice. *Diabetes.* 2003;52(2):434-41.
7. Leong HS, Grist M, Parsons H, Wambolt RB, Lopaschuk GD, Brownsey R, Allard MF. Accelerated rates of glycolysis in the hypertrophied heart: are they a methodological artifact? *Am J Physiol Endocrinol Metab.* 2002;282(5):E1039-45.
8. Neely JR, Denton RM, England PJ, Randle PJ. The effects of increased heart work on the tricarboxylate cycle and its interactions with glycolysis in the perfused rat heart. *Biochem J.* 1972;128(1):147-59.
9. Katz J, Dunn A. Glucose-2-t as a tracer for glucose metabolism. *Biochemistry.* 1967;6(1):1-5.
10. Le Belle JE, Harris NG, Williams SR, Bhakoo KK. A comparison of cell and tissue extraction techniques using high-resolution ^{1}H-NMR spectroscopy. *NMR Biomed.* 2002;15(1):37-44.
11. Santos CX, et al. Targeted redox inhibition of protein phosphatase 1 by Nox4 regulates eIF2alpha-mediated stress signaling. *EMBO J.* 2016;35(3):319-34.
12. Peterson JR, et al. Genetic silencing of Nox2 and Nox4 reveals differential roles of these NADPH oxidase homologues in the vasopressor and dipsogenic effects of brain angiotensin II. *Hypertension.* 2009;54(5):1106-14.
13. Gibbs C. The cytoplasmic phosphorylation potential. Its possible role in the control of myocardial respiration and cardiac contractility. *J Mol Cell Cardiol.* 1985;17(8):727-31.
14. Saupe KW, Spindler M, Tian R, Ingwall JS. Impaired cardiac energetics in mice lacking muscle-specific isoenzymes of creatine kinase. *Circ Res.* 1998;82(8):898-907.
15. Palmer JW, Tandler B, and Hoppel CL. Biochemical properties of subsarcolemmal and interfibrillar mitochondria isolated from rat cardiac muscle. *J Biol Chem.* 1977;252(23):8731-9.

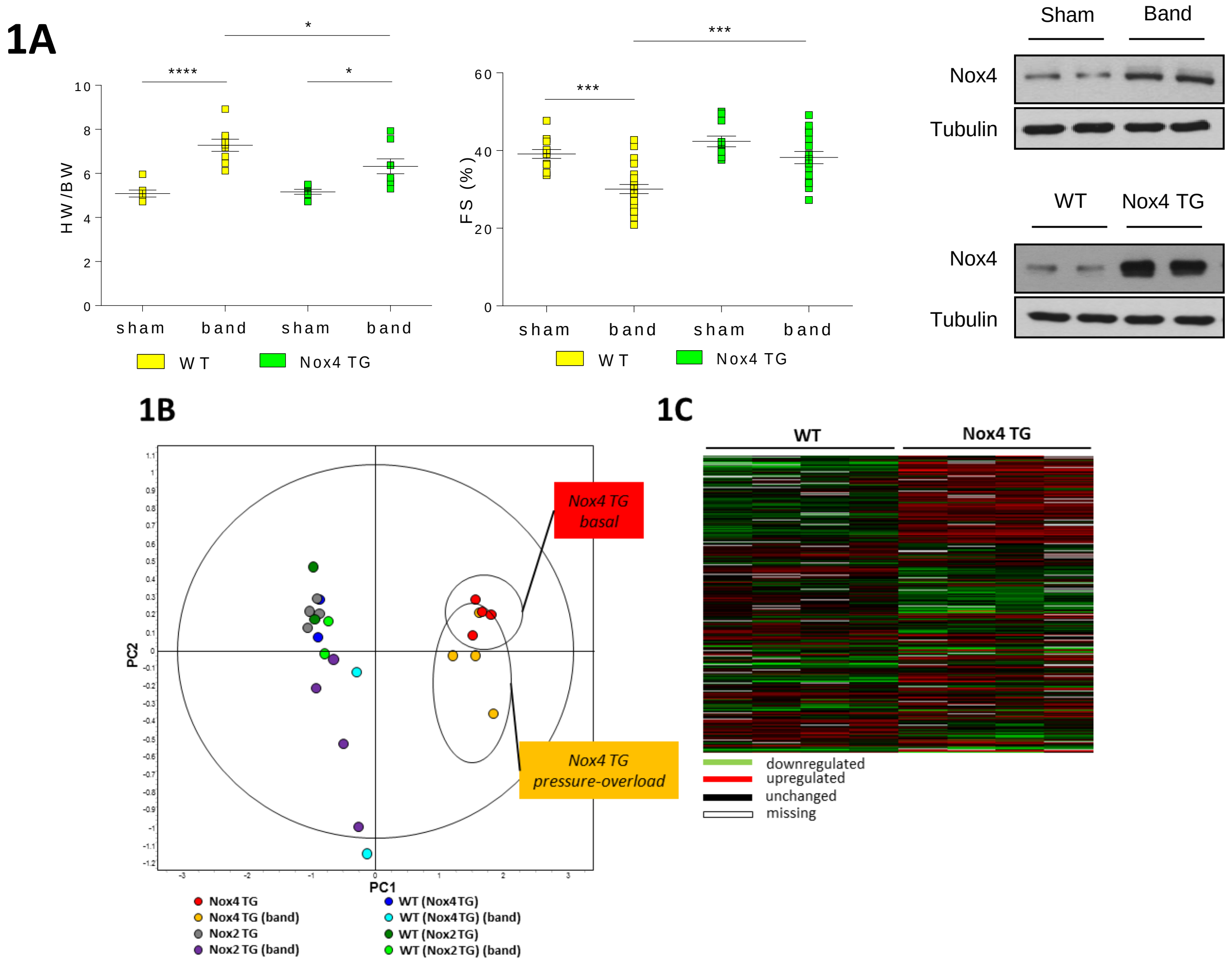

1D

Glycolytic and b-oxidative proteins	Fold change Nox4 TG vs WT *Basal*	Fold change Nox4 TG vs WT *Stress*
Fructose-bisphosphate aldolase A (Aldoa)	-2.64	-2.28
Triosephosphate isomerase (Tpi)	+1.35	+1.36
Phosphoglycerate mutase 1 (Pgam1)	+1.82	+1.76
β-enolase (Eno3)	-1.9	-1.73
Pyruvate kinase (Pkm)	+1.42	+1.36
Acyl CoA dehydrogenase, long-chain (Acadl)	-1.3	-1.34
Acyl CoA dehydrogenase, medium-chain (Acadm)	-1.36	-1.35
3-hydroxyacyl CoA dehydrogenase (Hadh)	-1.39	-1.3
β-ketoacyl-CoA thiolase (Acaa2)	-1.29	-1.21

Supplemental Figure 1. Comparison of the cardiac proteome of Nox4 TG and wild-type (WT) littermates. (**A**) Cardiac hypertrophy (n = 7-9 per group) and function (n ≥ 12) in Nox4 TG and WT mice 3 weeks after aortic banding. FS, echocardiographic fractional shortening. *$P < 0.05$; ***$P < 0.001$; ****$P < 0.0001$, 2-way ANOVA with Tukey's post hoc correction. Immunoblots to the right show the changes in Nox4 levels after banding as well as the difference in Nox4 levels between WT and Nox4 TG. (**B**) Principal component analysis of the proteome profile for Nox4 TG vs Nox2 TG vs WT, with and without pressure-overload stress. The contributions of the two principal components driving differential expression were 60.4% for PC1 and 7% for PC2. (**C**) Heat map of the cardiac proteome profile in Nox4 TG and WT, based on 2D difference in-gel electrophoresis dataset. n = 4 per group. (**D**) Metabolic proteins identified as statistically significantly altered in Nox4 TG vs WT hearts. The fold-change in abundance is reported under unstressed conditions and after aortic banding (Stress). *t* test, n = 4 per group.

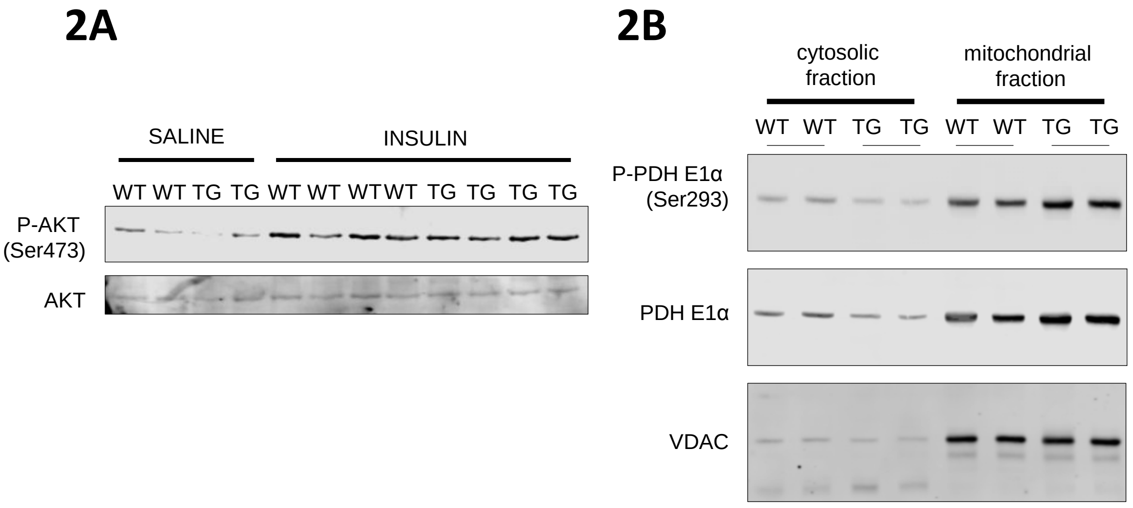

Supplemental Figure 2. Insulin sensitivity and PDH activity in Nox4 TG hearts. (**A**) Representative immunoblots showing levels of Akt phosphorylated at Ser473 and total Akt levels in heart tissue after treatment with i.p. insulin (0.75 IU/kg) or saline. Relates to Fig. 2E. (**B**) Representative immunoblots showing phosphorylated pyruvate dehydrogenase (P-Pdha E1α) and total Pdha E1α levels in mitochondrial fractions from WT and Nox4 TG hearts. Immunoblotting for VDAC was used as a mitochondrial-specific protein. Relates to Fig. 2F.

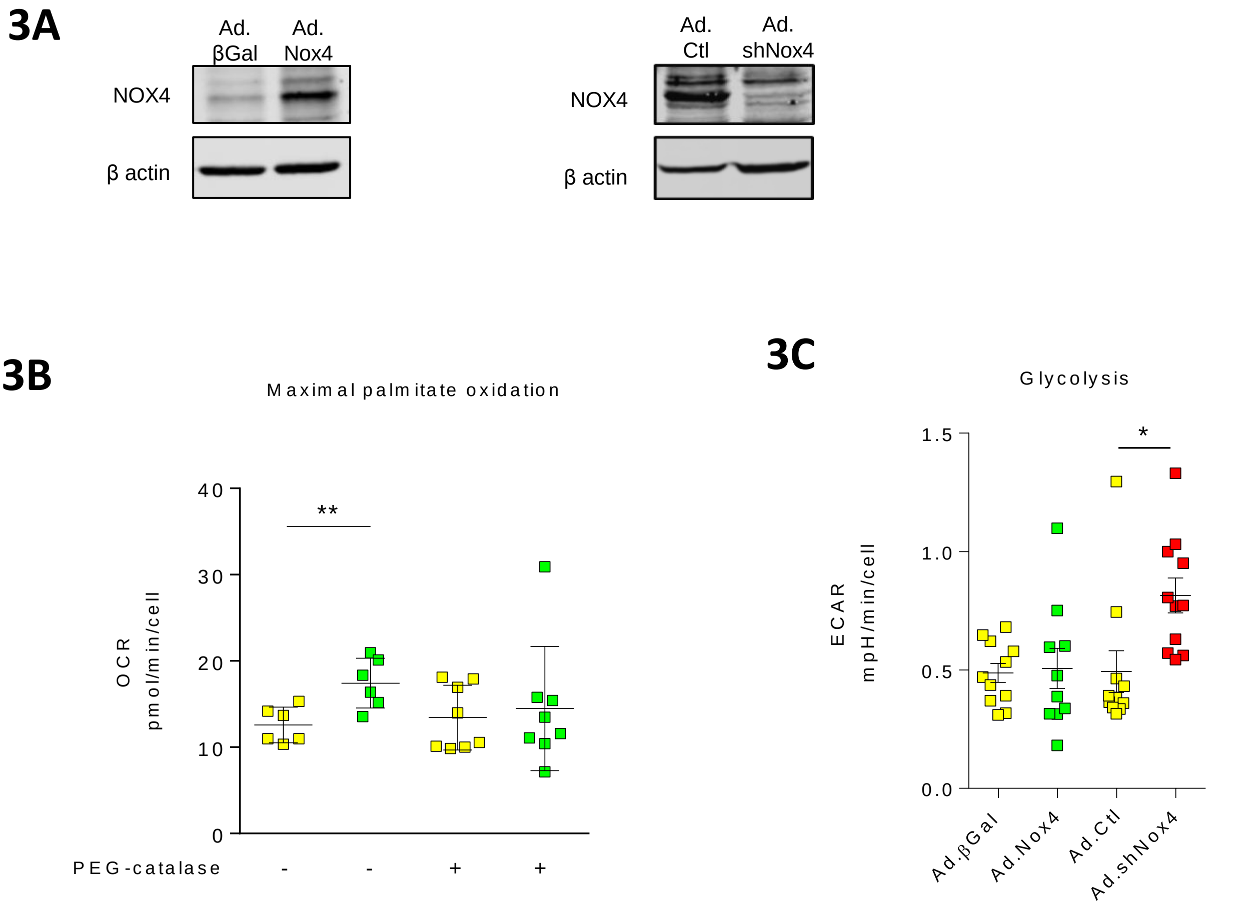

Supplemental Figure 3. Effect of change in Nox4 levels in cultured cardiomyocytes on fatty acid oxidation rate and glycolysis. (**A**) Representative immunoblots showing effect on Nox4 levels in cultured cardiomyocytes of adenoviral-mediated overexpression of Nox4 (Ad.Nox4) or a control β-galactosidase protein (Ad.β-Gal) (left panel) or shRNA-mediated knockdown of Nox4 (Ad.shNox4) or control (Ad.Ctl) (right panel). (**B**) Maximal oxygen consumption rate (OCR) for palmitate measured in the presence or absence of PEG-catalase (500U/mL) to degrade H_2O_2. $^{**}P < 0.01$, *t* test, n = 6-8 per group. (**C**) Effect of Nox4 overexpression or knockdown on glycolysis, assessed as the extracellular acidification rate (ECAR). $^{*}P < 0.05$, *t* test, n = 11 per group. OCR and extracellular acidification were measured in an extracellular flux analyser. Relates to Fig. 4.

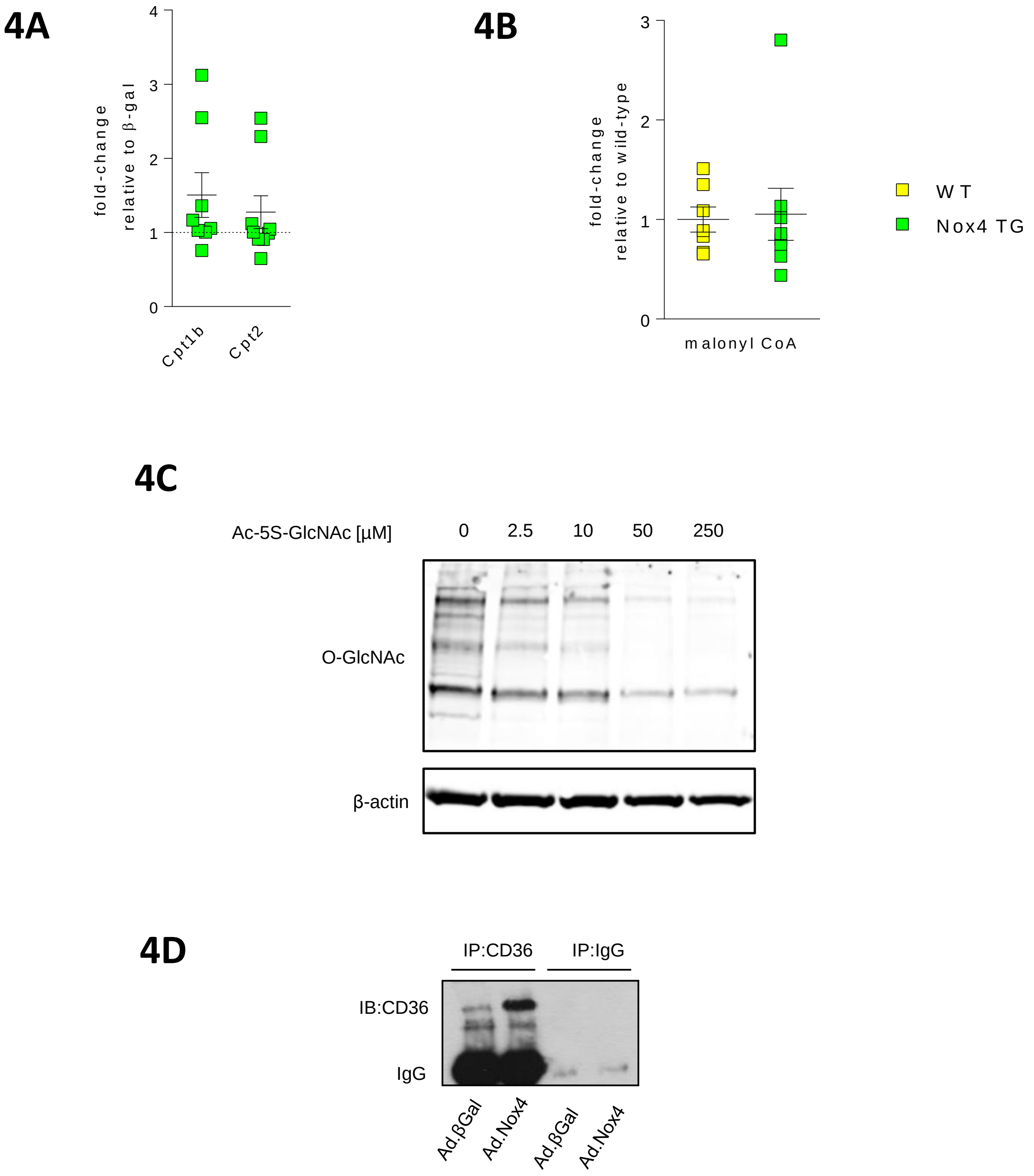

Supplemental Figure 4. Nox4 modulates fatty acid oxidation via protein O-GlcNAcylation. (**A**) Effect of Nox4 overexpression on mRNA levels of Cpt1b and Cpt2 in primary cultured cardiomyocytes. (**B**) Malonyl CoA levels in WT compared to Nox4 TG hearts (t-test, n=7 per group). (**C**) Immunoblot showing the dose-dependent inhibition of protein of O-GlcNAcylation by a specific HBP inhibitor, Ac-5S-GlcNAc, in primary cardiomyocytes. (**D**) Immunoblot demonstrating the specificity of the CD36 antibody used for IP as compared to non-specific IgG. Relates to Fig. 5.

5A

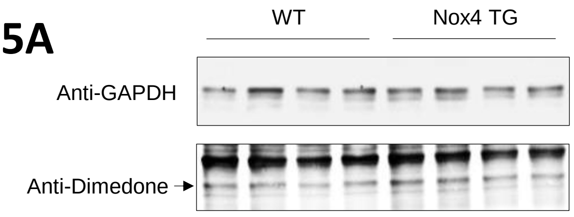

5B

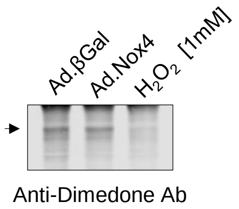

5C

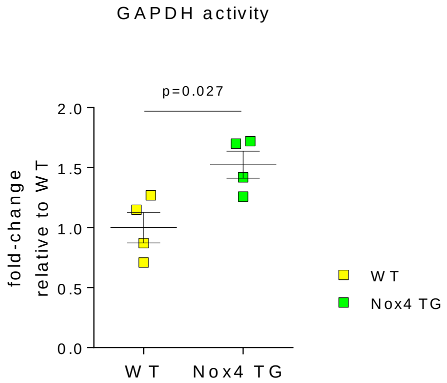

Supplemental Figure 5. Lack of inhibition of GAPDH by Nox4. (**A**) Immunoblot for anti-dimedone detection of oxidized GADPH in Nox4 TG and WT hearts. There was no difference between the groups. (**B**) Immunoblot for anti-dimedone detection of oxidized GADPH in cardiomyocytes infected with Ad.Nox4 or Ad.βGal control. Nox4 had no effect as compared to β-Gal. Treatment with H_2O_2 was used as a control and markedly reduced the dimedone signal, indicating hyperoxidation of GAPDH. (**C**) GADPH activity in Nox4 TG and WT hearts. *t* test, n = 4.

6A

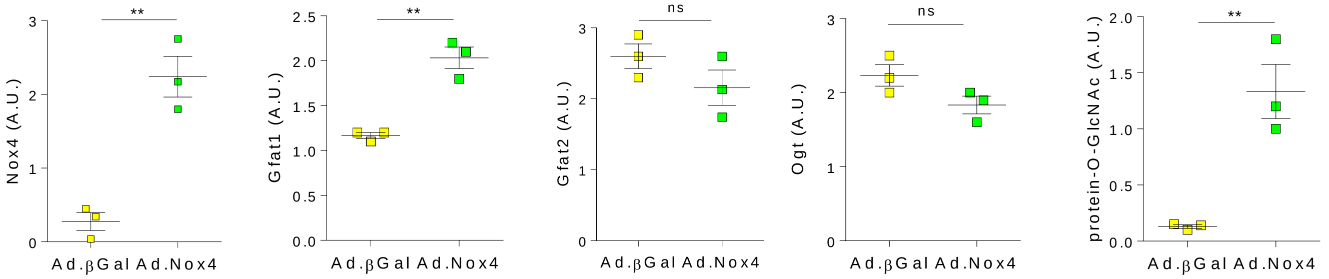
Nox4 (A.U.)
Gfat1 (A.U.)
Gfat2 (A.U.)
Ogt (A.U.)
protein-O-GlcNAc (A.U.)
**
**
ns
ns
**
Ad.βGal Ad.Nox4
Ad.βGal Ad.Nox4
Ad.βGal Ad.Nox4
Ad.βGal Ad.Nox4
Ad.βGal Ad.Nox4

6B

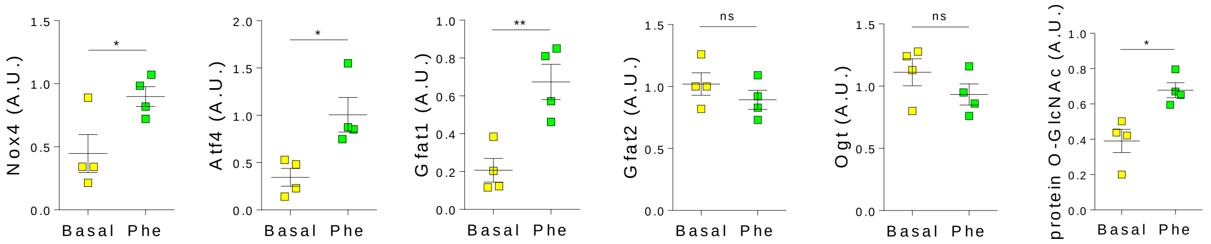
Nox4 (A.U.)
Atf4 (A.U.)
Gfat1 (A.U.)
Gfat2 (A.U.)
Ogt (A.U.)
protein O-GlcNAc (A.U.)
*
*
**
ns
ns
*
Basal Phe
Basal Phe
Basal Phe
Basal Phe
Basal Phe
Basal Phe

6C

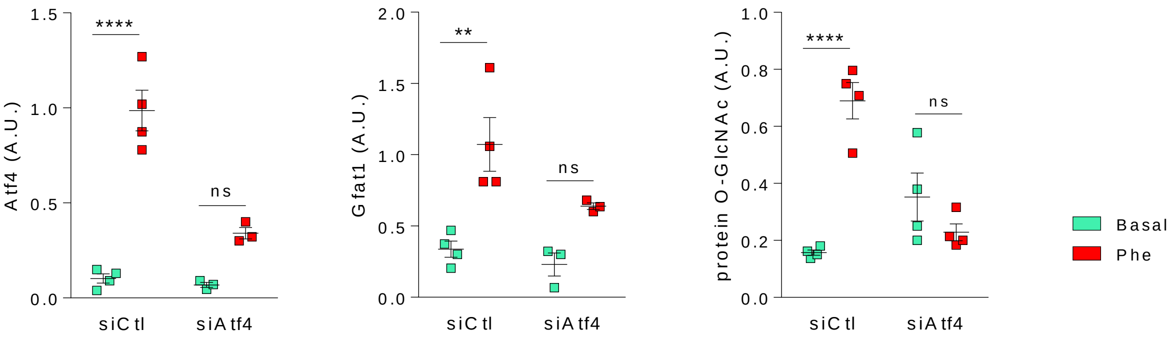
Atf4 (A.U.)
Gfat1 (A.U.)
protein O-GlcNAc (A.U.)
****
ns
**
ns
****
ns
siCtl siAtf4
siCtl siAtf4
siCtl siAtf4
Basal
Phe

6D

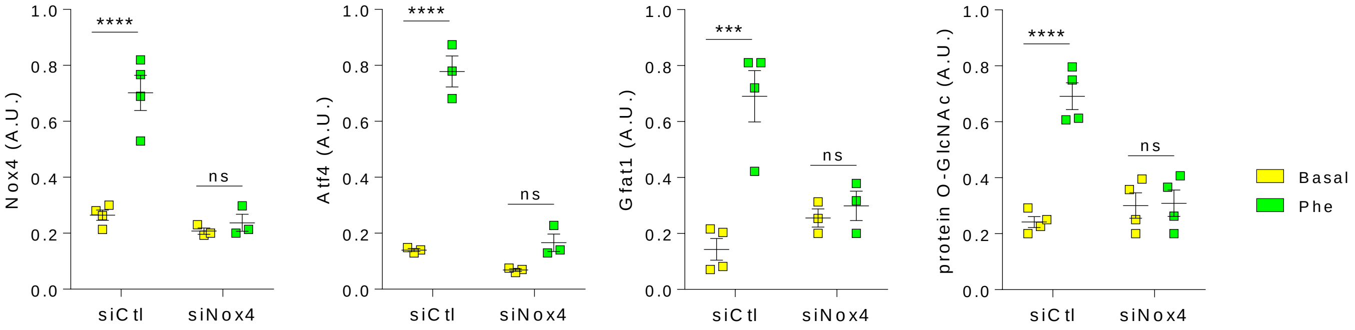
Nox4 (A.U.)
Atf4 (A.U.)
Gfat1 (A.U.)
protein O-GlcNAc (A.U.)
****
ns
****
ns
***
ns
****
ns
siCtl siNox4
siCtl siNox4
siCtl siNox4
siCtl siNox4
Basal
Phe

6E

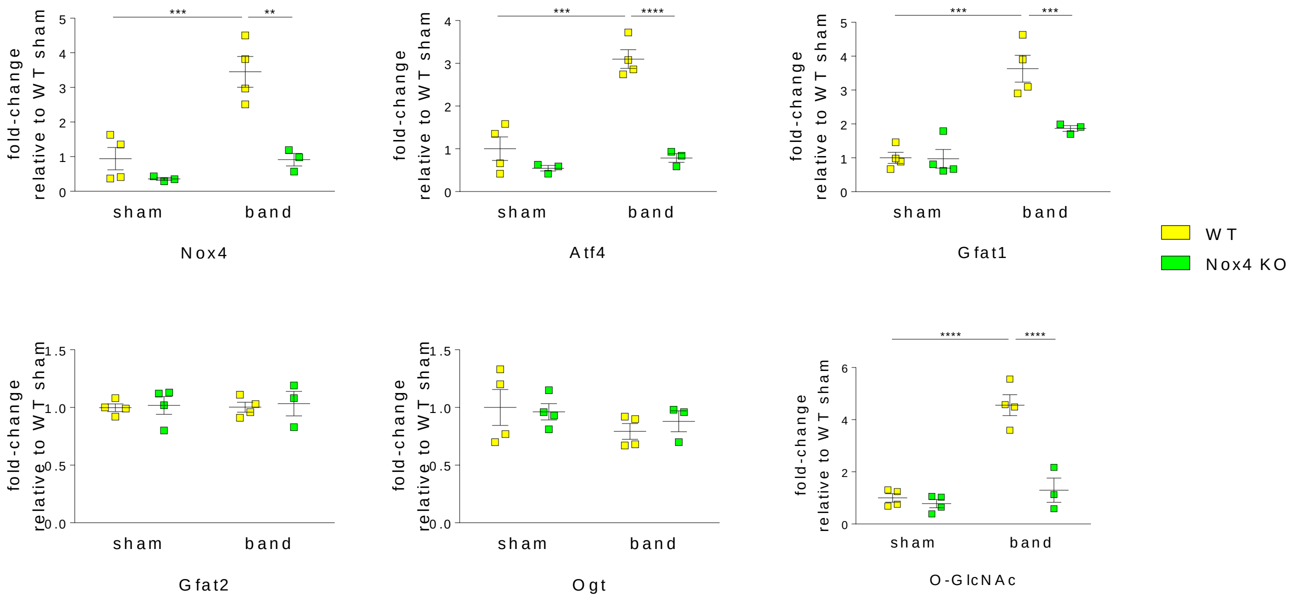

Supplemental Figure 6. Nox4 regulates ATF4-Gfpt1-O-GlcNAc signaling (relates to Fig. 6). (**A**) Mean data for the experiment illustrated in Fig. 6B. $**P < 0.01$, t test, n = 3. (**B**) Mean data for the experiment illustrated in Fig. 6D. Phe, phenylephrine. $*P < 0.05$; $**P < 0.01$; t test; n= 4. (**C**) Mean data for the experiment illustrated in Fig. 6E. $**P < 0.01$; $****P < 0.0001$, 2-way ANOVA with Tukey's post hoc correction, n =3-4 per group. (**D**) Mean data for the experiment illustrated in Fig. 6F. $***P < 0.001$; $****P < 0.0001$, 2-way ANOVA with Tukey's post hoc correction, n = 3-4 per group. (**E**) Mean data for the experiment illustrated in Fig. 6G. ***P < 0.001; ****P < 0.0001, 2-way ANOVA with Tukey's post hoc correction, n = 3-4 per group.

7A

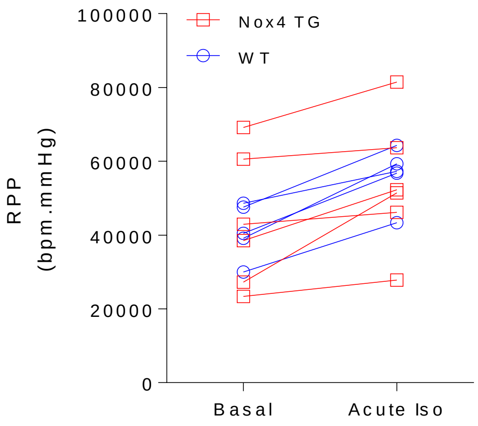

7B

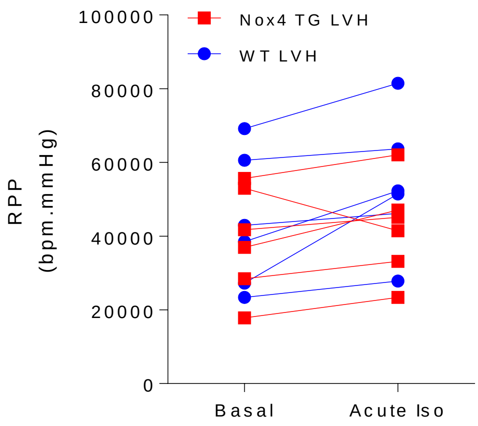

Supplemental Fig. 7. Effects of Nox4 on cardiac energetic state. Rate pressure product (RPP) of WT and Nox4 TG hearts during the cardiac energetic studies reported in Fig. 7, A-D. Panel **A** shows data for non-hypertrophied hearts and panel **B** for hypertrophied hearts. Acute Iso denotes acute isoproterenol challenge. *t* test, n = 6 per group.
